# Supplementary material for: Antiviral Immune Response as a Trigger of FUS Proteinopathy in Amyotrophic Lateral Sclerosis
Source: Cell Rep. 2019 Dec 24;29(13):4496–4508.e4. doi: 10.1016/j.celrep.2019.11.094 (PMC6941233; doi:10.1016/j.celrep.2019.11.094)
Supplement: Document S1. Figures S1–S7 and Table S1 [file mmc1.pdf]

Cell Reports, Volume *29*

Supplemental Information

Antiviral Immune Response as a Trigger of FUS Proteinopathy in Amyotrophic Lateral Sclerosis

Tatyana A. Shelkovnikova, Haiyan An, Lucy Skelt, John S. Tregoning, Ian R. Humphreys, and Vladimir L. Buchman

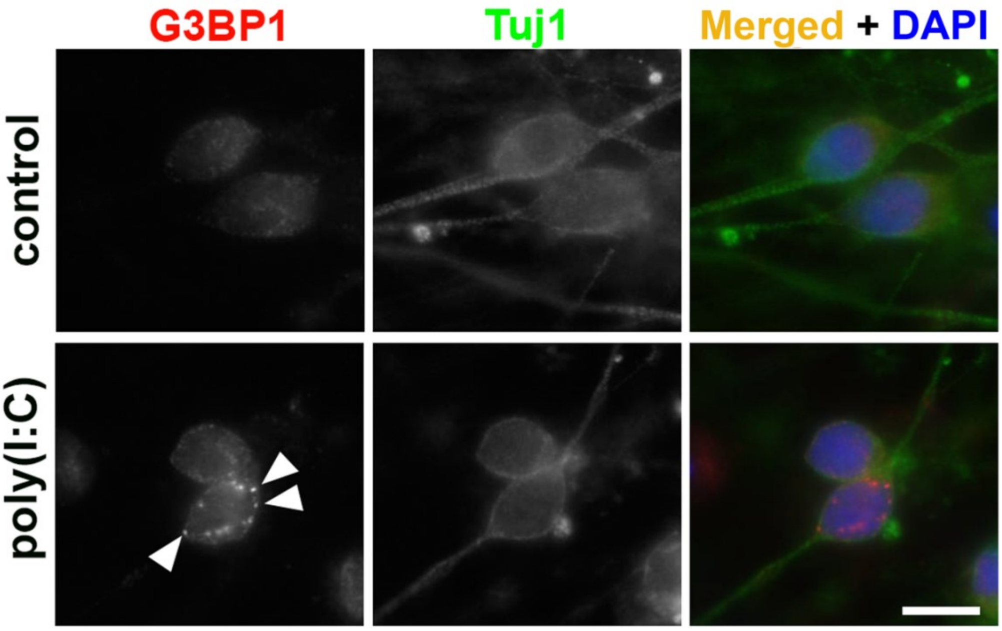

Figure S1. Poly(I:C) transfection is capable of inducing SGs in human embryonic stem (ES) cell derived motor neurons, Related to Figure 1.

DIV40 neurons were analysed 8 h post-transfection with poly(I:C). Arrowheads point to G3BP1-positive SGs. Scale bar, 10 µm.

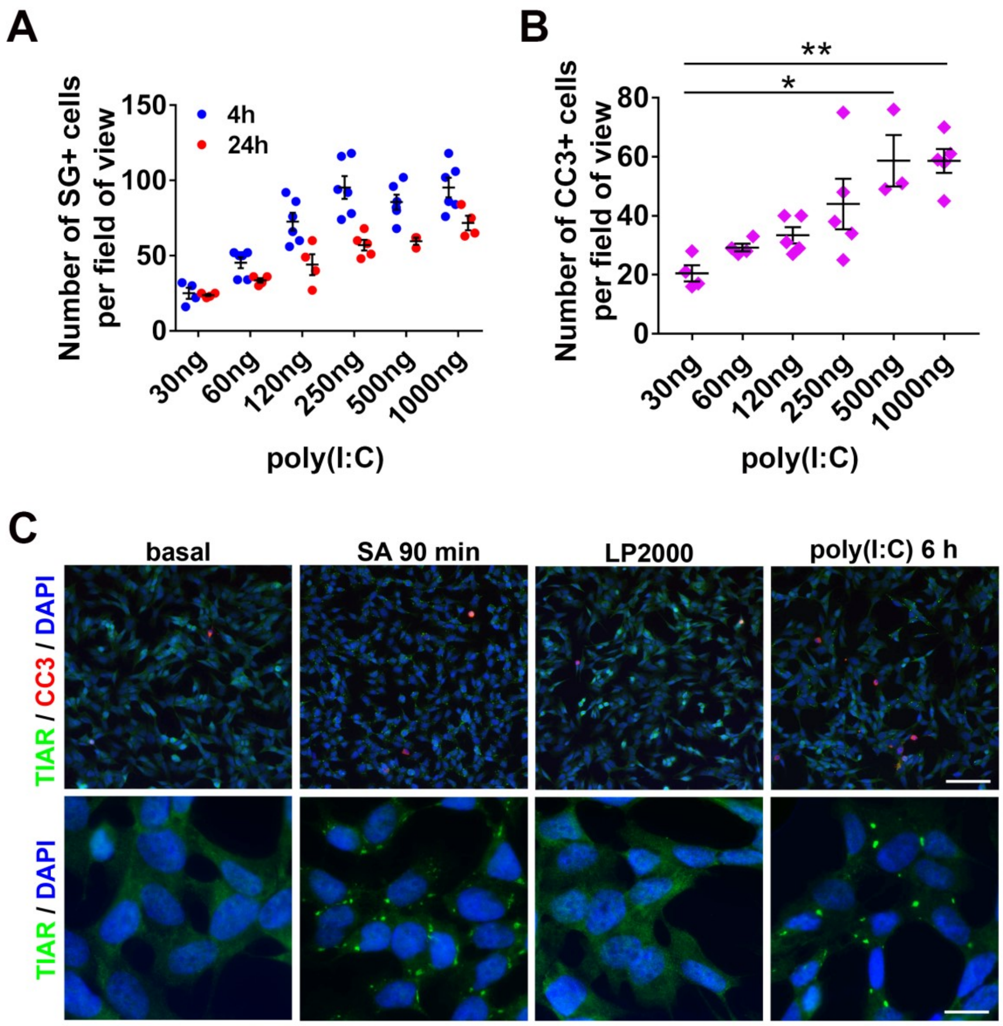

Figure S2. Concentration-dependent ability of poly(I:C) to induce SGs and toxicity in SH-SY5Y cells, Related to Figure 1.

(A) Concentration-dependent induction of SGs by poly(I:C) in SH-SY5Y cells. Cells were transfected with the corresponding amount of poly(I:C) (per well, in 24-well plates) and analysed 4 and 24 h post-transfection using anti-TIAR staining (x40 magnification). Data are represented as mean±SEM.

(B) Concentration-dependent toxicity of poly(I:C) in SH-SY5Y cells. Cells were transfected with the corresponding amount of poly(I:C) (per well, in 24-well plates) and analysed 24 h post-transfection using anti-cleaved caspase 3 (CC3) staining (x20 magnification). Note that 250 ng was selected for all subsequent experiments due to the optimal balance between SG induction and cell survival. Data are represented as mean±SEM. *$p<0.05$, **$p<0.01$ (Mann-Whitney *U* test).

(C) Poly(I:C) stimulation for 6 h and SA treatment for 1.5 h do not affect cell morphology and do not induce significant toxicity. SH-SY5Y cells were treated with SA or transfected with poly(I:C) and analysed after 1.5 h or 6 h, respectively, using a combination of anti-TIAR and cleaved caspase 3 (CC3) staining. Lipofectamine2000 (LP2000)-treated cells were included as a control for poly(I:C) transfection. Scale bars, general plane – 100 μm, close up – 10 μm.

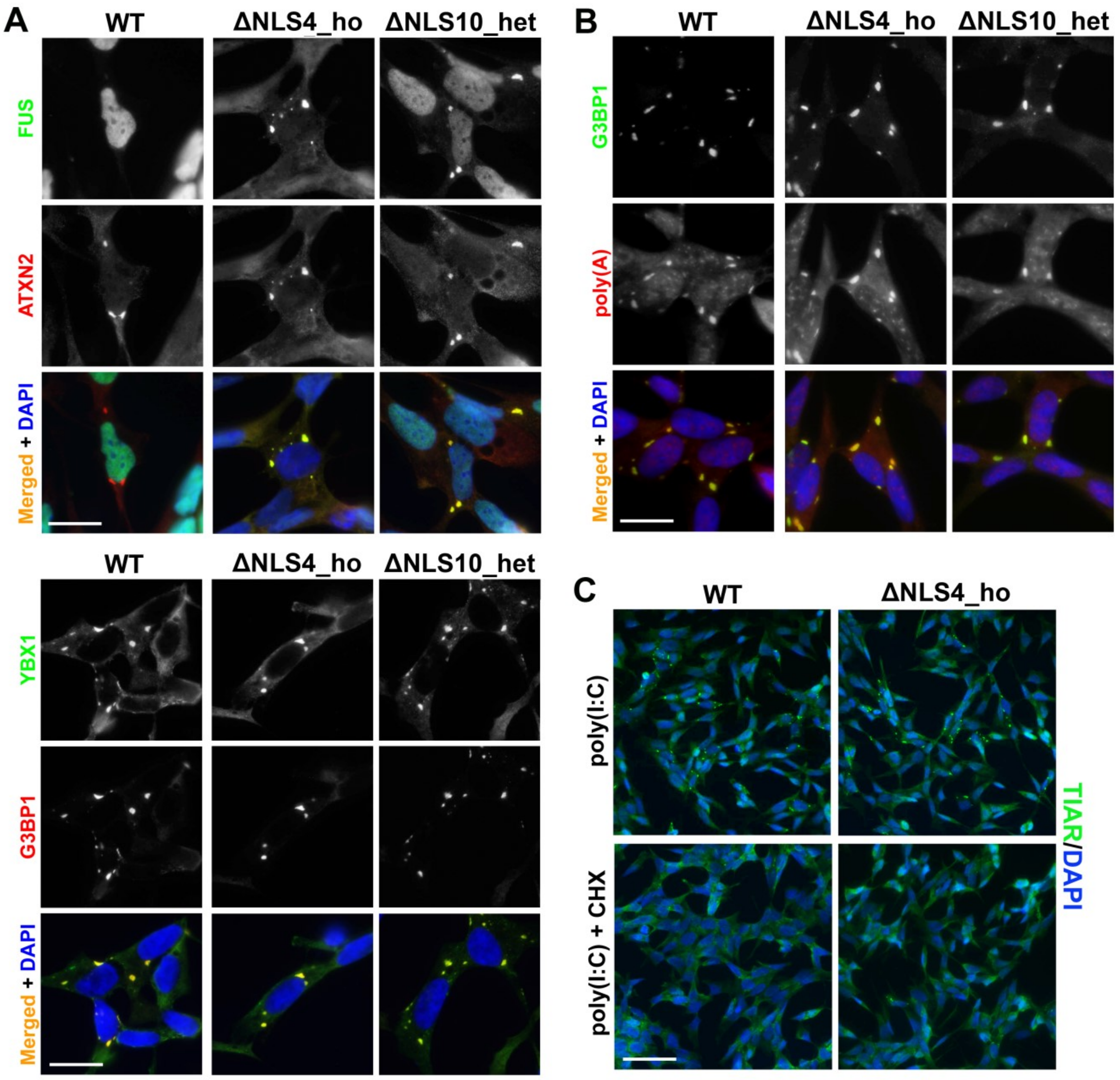

Figure S3. Poly(I:C)-induced SGs, including those in FUSΔNLS cells, contain SG markers ATXN2 and YBX1 (A) as well as polyadenylated RNA (B), and are sensitive to cycloheximide treatment (C), Related to Figure 2.

Cell lines were transfected with poly(I:C) and analysed 4 h post-transfection. In C, cycloheximide (CHX) was added 1 h prior poly(I:C) transfection. Representative images are shown. Scale bar, A, B – 10 µm, C – 50 µm.

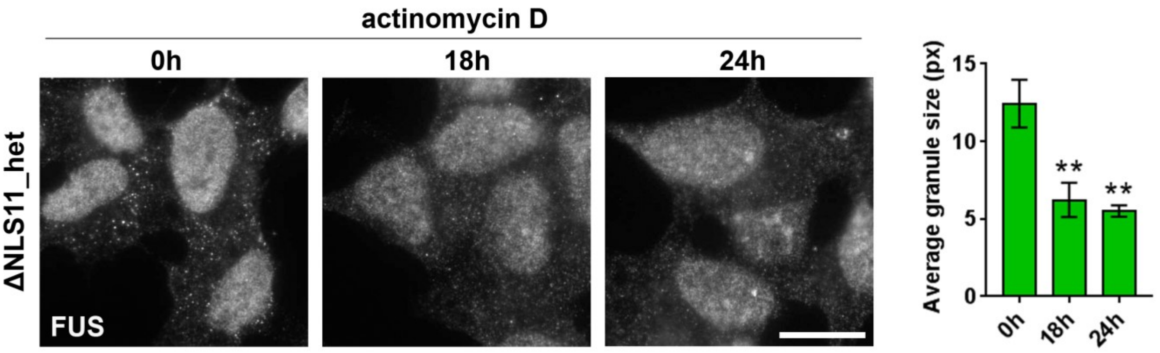

Figure S4. Spontaneous FUS granules formed by endogenous mutant FUS protein (endoFGs) are sensitive to transcription inhibition, Related to Figure 3.

ΔNLS11_het line which possesses endoFGs was treated with actinomycin D for 18 h and 24 h, and the size of FGs was measured using Image J (px – pixels). Data are represented as mean±SEM. **p<0.01 (ANOVA with Dunn's test). Scale bar, 10 μm.

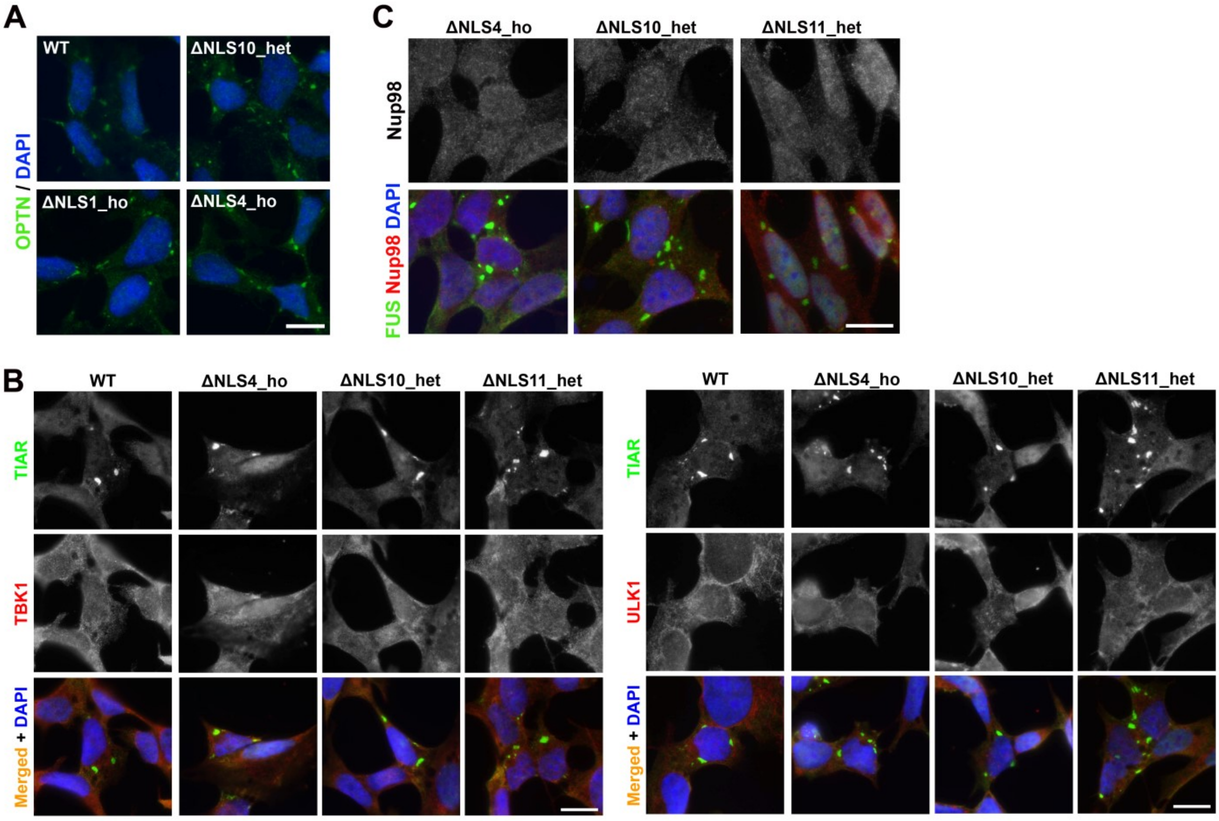

Figure S5. Analysis of sequestration of autophagy-related proteins and NPC factors into mutant FUS assemblies, Related to Figure 4.

(A) Optineurin is recruited into SA-induced SGs in WT and FUSΔNLS cells. Cells were treated with SA for 1 h.

(B) Optineurin interactor TBK1 and the primary component of the autophagy initiation complex ULK1 are not detectable in mutant FUS-containing cytoplasmic assemblies in poly(I:C)-stimulated FUSΔNLS cells.

(C) Nucleocytoplasmic transport factor Nup98 is not detected in mutant FUS-containing cytoplasmic assemblies in poly(I:C)-stimulated FUSΔNLS cells.

In B and C, cells were analysed 6 h post-transfection; note that ΔNLS11_het cell line contains endoFGs and therefore forms FAs not SGs.

Scale bars, 10 μm.

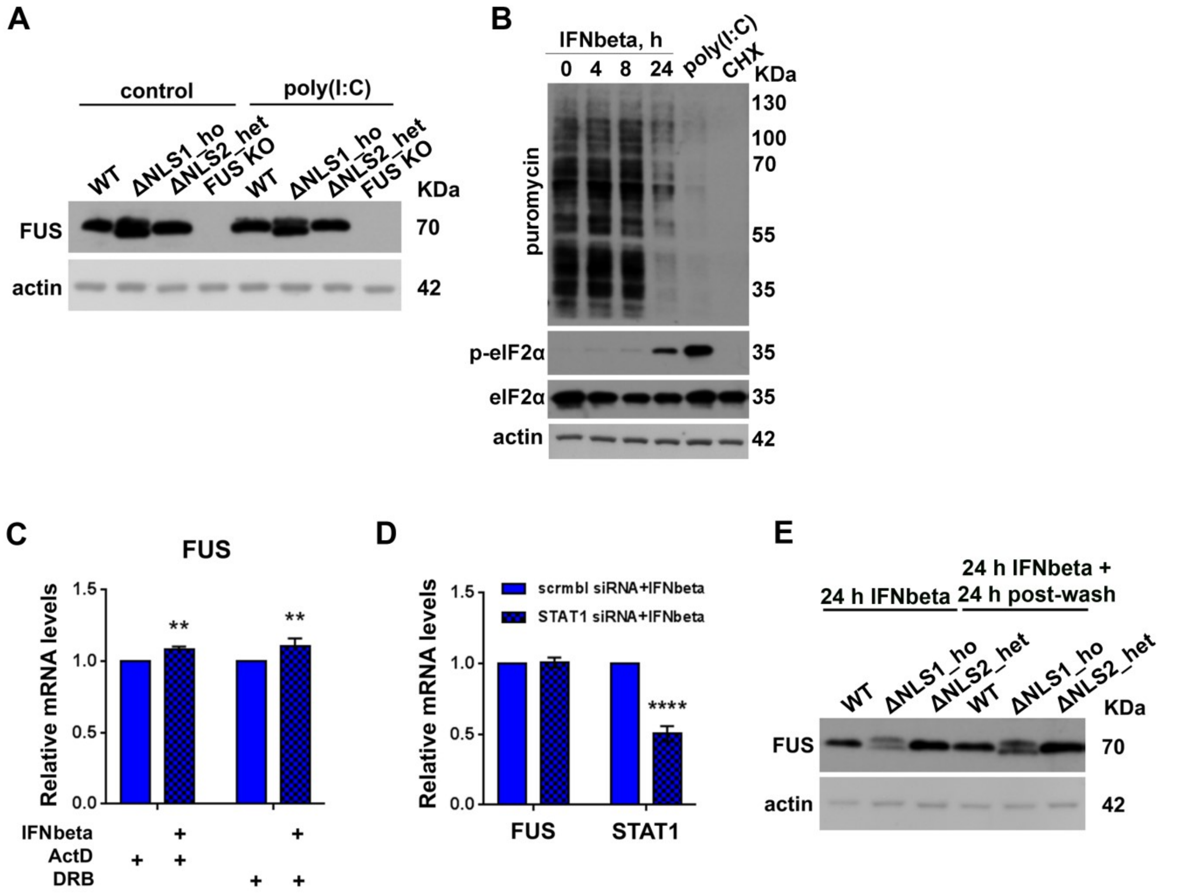

Figure S6. IFN-beta causes accumulation of FUS protein, Related to Figure 6.

(A) Poly(I:C) transfection does not result in increased FUS protein level in WT or FUSΔNLS cells. Cells were analysed at 24 h post-transfection.

(B) Puromycin labelling of proteins reveals significant impairment of protein translation due to eIF2α phosphorylation in poly(I:C)-transfected cells but not in IFN-beta treated cells. Cells were treated with IFN-beta and collected at the indicated time-points, or collected 8 h after poly(I:C) transfection. Cells treated with a protein synthesis inhibitor cycloheximide (CHX) were included as a negative control.

(C) Transcription inhibition does not prevent IFN-beta induced FUS mRNA accumulation. Cells were pre-treated with transcription inhibitors actinomycin D or DRB for 1 h. FUS mRNA level was measured 4 h into IFN-beta treatment by qRT-PCR. Data are represented as mean±SEM. N=4, **p<0.01 (Mann-Whitney *U* test).

(D) FUS mRNA upregulation in IFN-beta treated cells is independent of STAT1. Cells were transfected with scrambled siRNA or STAT1 siRNA; 48 h post-transfection, cells were subjected to IFN-beta for 4 h, and FUS mRNA level was measured by qRT-PCR. Data are represented as mean±SEM. N=4, ****p<0.0001 (Mann-Whitney *U* test).

(E) FUS protein continues to accumulate after IFN-beta wash-off both in WT and FUSΔNLS cells. Cells were treated with IFN-beta for 24 h, washed and analysed after another 24 h.

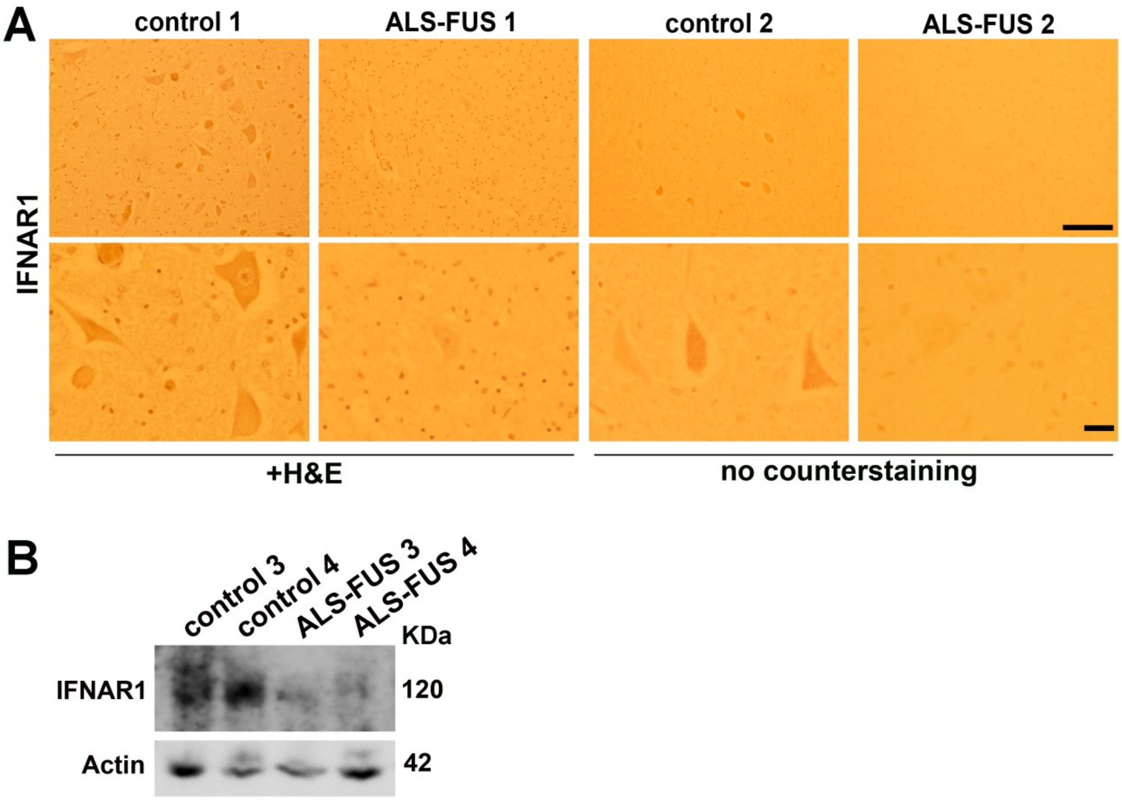

Figure S7. IFNAR1, one of the two IFN receptor (IFNAR) subunits, is highly expressed in spinal motor neurons and is downregulated in ALS-FUS, Related to Figure 6.

(A) IFNAR1 immunohistochemistry in the spinal cord sections for two control cases and two ALS-FUS cases. Note that control 1 and ALS-FUS 1 were counter-stained with H&E. Scale bars, 50 μm and 10 μm in upper and lower panels, respectively.

(B) Western blot analysis of IFNAR1 levels in the spinal cord of two control and two ALS-FUS patients.

Table S1. Primers for qRT-PCR used in the study, Related to STAR Methods.

Target and primer sequence	Source	ID
GAPDH, forward primer: 5’-TCGCCAGCCGAGCCA-3’	An et al., 2019	N/A
GAPDH, reverse primer: 5’-GAGTTAAAAGCAGCCCTGGTG -3’	An et al., 2019	N/A
FUS, forward primer: 5’-GGAACTCAGTCAACTCCCCA-3’	An et al., 2019	N/A
FUS, reverse primer: 5’-TACCGTAACTTCCCGAGGTG-3’	An et al., 2019	N/A
FUS ex7-, forward primer: 5’-CAGAGGTGGCATGGGGC-3'	This paper	N/A
FUS ex7-, reverse primer: 5’-TGTAACATTCTCACCCAGGC-3	This paper	N/A
FUS pre-mRNA, forward primer1: 5’-GAACCACCTCCAGAAAGGGG-3’	This paper	N/A
FUS pre-mRNA, reverse primer1: 5’-TGGGGCAAACCCATTTGGTA-3’	This paper	N/A
FUS pre-mRNA, forward primer2: 5’- GAAGCCGCGGAGAAGAGTAA-3’	This paper	N/A
FUS pre-mRNA, reverse primer2: 5’- AAGAAAAGACTTCCCGCCCC-3’	This paper	N/A
STAT1, forward primer: 5’-CTGTGCGTAGCTGCTCCTTT-3’	This paper	N/A
STAT1, reverse primer: 5’-GGTGAACCTGCTCCAGGAAT-3’	This paper	N/A
IFN-beta, forward primer: 5'-ACGCCGCATTGACCATCTAT-3'	This paper	N/A
IFN-beta, reverse primer: 5’-AGCCAGGAGGTTCTCAACAA-3’	This paper	N/A
IFIT3, forward primer: 5’-AGAGACACAGAGGGCAGTCA-3'	This paper	N/A
IFIT3, reverse primer: 5’-AAGTTCCAGGTGAAATGGCA-3’	This paper	N/A
CXCL10, forward primer: 5’-AAGTTCCAGGTGAAATGGCA-3’	This paper	N/A
CXCL10, reverse primer: 5’-ATGCTGATGCAGGTACAGCG-3’	This paper	N/A
CHOP, forward primer: 5’-TTAAAGATGAGCGGGTGGC-3’	Shelkovnikova et al., 2017	N/A
CHOP, reverse primer: 5’-GCTTTCAGGTGTGGTGATGTA-3’	Shelkovnikova et al., 2017	N/A
