# Supplementary material for: Antiviral Immune Response as a Trigger of FUS Proteinopathy in Amyotrophic Lateral Sclerosis
Source: Cell Rep. 2019 Dec 24;29(13):4496–4508.e4. doi: 10.1016/j.celrep.2019.11.094 (PMC6941233; doi:10.1016/j.celrep.2019.11.094)
Supplement: Document S2. Article plus Supplemental Information [file mmc3.pdf]

Cell Reports

Article

Antiviral Immune Response as a Trigger of FUS Proteinopathy in Amyotrophic Lateral Sclerosis

Graphical Abstract

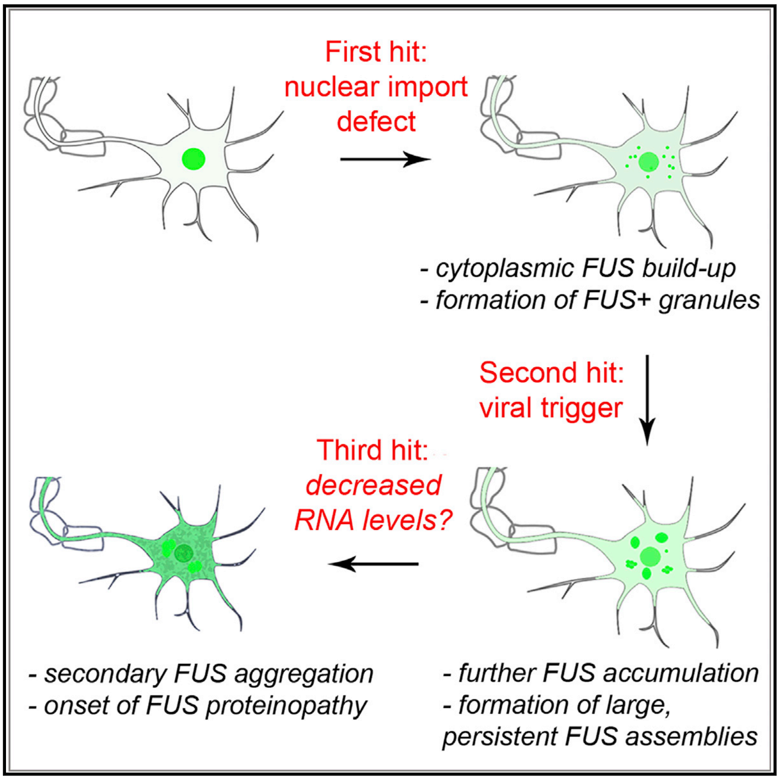

Highlights

- Viral infection or its mimic induce large, persistent assemblies of mutant FUS
- These assemblies sequester optineurin and nucleocytoplasmic transport factors
- Mutant FUS-expressing cells are hypersensitive to virus-induced toxicity
- Type I interferon induced by viral infections promotes accumulation of FUS protein

Authors

Tatyana A. Shelkovnikova, Haiyan An, Lucy Skelt, John S. Tregoning, Ian R. Humphreys, Vladimir L. Buchman

Correspondence

shelkovnikovat@cardiff.ac.uk (T.A.S.), buchmanvl@cardiff.ac.uk (V.L.B.)

In Brief

Amyotrophic lateral sclerosis caused by mutations in the *FUS* gene is characterized by cytoplasmic FUS pathology (FUS proteinopathy). Shelkovnikova et al. find that the antiviral immune response promotes FUS protein accumulation and its coalescence into persistent cytoplasmic assemblies. Viral infection can serve as a trigger of FUS proteinopathy in ALS.

Shelkovnikova et al., 2019, Cell Reports *29*, 4496–4508
December 24, 2019 © 2019 The Author(s).
https://doi.org/10.1016/j.celrep.2019.11.094

CellPress

Cell Reports
Article

Antiviral Immune Response as a Trigger of FUS Proteinopathy in Amyotrophic Lateral Sclerosis

Tatyana A. Shelkovnikova,[1,2,6,7,*] Haiyan An,[1,2,6] Lucy Skelt,[1] John S. Tregoning,[3] Ian R. Humphreys,[4] and Vladimir L. Buchman[1,5,*]
[1]Biomedicine Division, School of Biosciences, Cardiff University, Cardiff CF10 3AX, UK
[2]Medicines Discovery Institute, Cardiff University, Cardiff CF10 3AT, UK
[3]Department of Infectious Disease, St Mary's Campus, Imperial College London, London W2 1PG, UK
[4]Systems Immunity Research Institute, School of Medicine, Cardiff University, Cardiff CF14 4XN, UK
[5]Institute of Physiologically Active Compounds of RAS, Chernogolovka 142432, Russian Federation
[6]These authors contributed equally
[7]Lead Contact
*Correspondence: shelkovnikovat@cardiff.ac.uk (T.A.S.), buchmanvl@cardiff.ac.uk (V.L.B.)
https://doi.org/10.1016/j.celrep.2019.11.094

SUMMARY

Mutations in the *FUS* gene cause familial amyotrophic lateral sclerosis (ALS-FUS). In ALS-FUS, FUS-positive inclusions are detected in the cytoplasm of neurons and glia, a condition known as FUS proteinopathy. Mutant FUS incorporates into stress granules (SGs) and can spontaneously form cytoplasmic RNA granules in cultured cells. However, it is unclear what can trigger the persistence of mutant FUS assemblies and lead to inclusion formation. Using CRISPR/Cas9 cell lines and patient fibroblasts, we find that the viral mimic dsRNA poly(I:C) or a SG-inducing virus causes the sustained presence of mutant FUS assemblies. These assemblies sequester the autophagy receptor optineurin and nucleocytoplasmic transport factors. Furthermore, an integral component of the antiviral immune response, type I interferon, promotes FUS protein accumulation by increasing FUS mRNA stability. Finally, mutant FUS-expressing cells are hypersensitive to dsRNA toxicity. Our data suggest that the antiviral immune response is a plausible second hit for FUS proteinopathy.

INTRODUCTION

Amyotrophic lateral sclerosis (ALS) is the most common form of motor neuron disease. It is characterized by selective loss of upper and lower motor neurons in the central nervous system (CNS), which causes weakness and paralysis of the skeletal muscles they control (Peters et al., 2015). Although most cases are sporadic ALS (sALS), ~10% of cases bear a familial ALS (fALS) component. Mutations in more than 25 genes have been proven to cause the disease, with the *FUS* gene being one of the major fALS-causative genes (Kwiatkowski et al., 2009; Vance et al., 2009).

FUS encodes a predominantly nuclear DNA/RNA binding protein with multiple functions in RNA metabolism (Ratti and Buratti, 2016). Most ALS-causative mutations affect the nuclear localization signal (NLS) of FUS on its C terminus, thereby disrupting nuclear import of the protein and causing its cytoplasmic overabundance (Bosco et al., 2010; Dormann et al., 2010). Patients with ALS caused by *FUS* mutations (ALS-FUS) present with cytoplasmic FUS-positive inclusions in neurons and glia (Deng et al., 2014). Inclusions formed by non-mutated FUS protein are also found in the brain of some frontotemporal lobar degeneration (FTLD) patients (atypical FTLD-U subtype) (Neumann et al., 2009). Thus, conditions characterized by the presence of abnormal FUS inclusions are collectively called FUS proteinopathies.

Although FUS readily aggregates in the test tube, this is not the case *in vivo*, and available rodent models expressing mutant FUS do not develop FUS aggregates in the CNS (Devoy et al., 2017; Huang et al., 2011; López-Erauskin et al., 2018; Scekic-Zahirovic et al., 2016; Sharma et al., 2016). Our studies showed that to achieve efficient FUS aggregation in the murine nervous system, highly aggregate-prone artificial variants of FUS lacking RNA binding domains have to be used (Robinson et al., 2015; Shelkovnikova et al., 2013a). Yet in FUS proteinopathies, full-length FUS or FUS with small C-terminal truncations forms cytoplasmic inclusions. This implies the requirement of an additional trigger that causes robust FUS aggregation and inclusion formation in human disease. Mutant FUS is characterized by a strong affinity to stress granules (SGs), a type of cytoplasmic stress-induced ribonucleoprotein (RNP) assembly (Bosco et al., 2010; Dormann et al., 2010). SGs have been heavily implicated in ALS pathogenesis, because ALS-causative mutations affect not only FUS but also several other SG proteins (Li et al., 2013). In addition to being recruited to stress-induced SGs, overexpressed mutant FUS can spontaneously form small cytoplasmic granules that coalesce into larger assemblies containing SG markers (Kino et al., 2011; Shelkovnikova et al., 2014a). It has been proposed that FUS-enriched cytoplasmic assemblies, when they persist, may serve as precursors of pathological FUS inclusions in FUS proteinopathies (Bentmann et al., 2013; Shelkovnikova et al., 2013b). Conspicuously, FUS-positive inclusions in ALS-FUS contain SG marker proteins (Dormann et al., 2010). However, the nature of a stressor (or stressors) that can induce sustained presence of FUS-positive assemblies and

 © 2019 The Author(s).
This is an open access article under the CC BY license (http://creativecommons.org/licenses/by/4.0/).

thereby act as a trigger, or second hit, in FUS proteinopathies remains experimentally unaddressed.

Epidemiological and clinical evidence for a connection between viral infection and ALS has been accumulating for decades (Celeste and Miller, 2018; Ravits, 2005; Vandenberghe et al., 2010). For example, it is known that individuals with a history of poliomyelitis have a higher risk of developing motor neuron disease later in life (Martyn et al., 1988). Similarly, patients infected with HIV or human T cell leukemia virus 1 develop neurological disorders resembling clinical features of ALS (Alfahad and Nath, 2013). Importantly, multiple viruses are able to induce SG assembly (McCormick and Khaperskyy, 2017; White and Lloyd, 2012).

In the current study, we show that the exposure to foreign double-stranded RNA (dsRNA), typical for some viral infections, is a potent inducer of persistent FUS-enriched assemblies in the cytoplasm of cells expressing either exogenous or endogenous mutant FUS. Furthermore, we show that type I interferon (IFN), the central component of antiviral signaling, promotes accumulation of FUS protein. We propose that the antiviral immune response, with its profound effect on FUS levels and distribution, can serve as a trigger of FUS proteinopathy in ALS-FUS.

RESULTS

Viral dsRNA Mimic Causes Formation of Persistent SGs that Recruit Mutant FUS

Our initial aim was to identify stressors that can induce the prolonged presence of SGs in normal (wild-type [WT]) human neuroblastoma (SH-SY5Y) cells. In our analysis, we included neurodegeneration-relevant stressors: sodium arsenite (SA, oxidative stress), dithiothreitol (DTT, endoplasmic reticulum [ER] stress), and MG132 (proteasome inhibition). In addition, we tested a combination of the heat shock protein (HSP) 70 inhibitor pifithrin-μ and puromycin that is known to induce SG formation by simultaneous polysome dissociation and accumulation of misfolded proteins (Bounedjah et al., 2014). Viral infection can be a potent SG inducer; therefore, a viral dsRNA mimic, synthetic dsRNA polyinosinic:polycytidylic acid (poly(I:C)), was included. It is proposed that repetitive stresses causing multiple cycles of SG assembly-disassembly might lead to the appearance of persistent SGs (Wolozin, 2012). Stress induced by SA, DTT, and MG132 is reversible, which allowed examination of the effect of repetitive stresses. The treatment timeline for each stressor is schematically depicted in Figure 1A. SGs were visualized by staining for the core SG protein G3BP1. SA, DTT, MG132, a combination of pifithrin-μ and puromycin, and poly(I:C) induced SG assembly in 100%, 95.3% ± 1.8%, 34.7% ± 2.3%, 30.0% ± 3.6%, and 52.4% ± 3.0% of cells, respectively (Figure 1B). Two consecutive stresses with SA, DTT, and MG132, separated by a 24 h recovery period, did not increase SG numbers, and the removal of the stressor after the first and second rounds of stress led to SG disassembly with equal efficiency (Figure 1B). The viral infection mimic was the only stressor whose single application induced the assembly of persistent SGs: 24 and 48 h after poly(I:C) transfection, SGs were still detectable in 47.6% ± 3.5% and 27.7% ± 4.0% of cells, respectively (Figure 1B). Interestingly, we found that poly(I:C) was also able to induce SGs in a fraction of human embryonic stem cell (hESC)-derived motor neurons (Figure S1). Given the epidemiological link between viral infection and ALS and the ability of multiple viruses to interact with the SG pathway, we focused on this stressor.

Several stressors are known to cause the formation of FUS-positive SGs in cells expressing mutant versions of the protein, including oxidative stress, heat shock, osmotic stress, and proteasome inhibition (Dormann et al., 2010; Mateju et al., 2017; Sama et al., 2013; Shelkovnikova et al., 2014a); however, the effect of viral infection on mutant FUS has not been reported. Synthetic dsRNA poly(I:C) is capable of triggering core features of the antiviral response upon its delivery into mammalian cells, including SG formation (Weissbach and Scadden, 2012). The use of poly(I:C) can mimic a response typical for multiple types and classes of viruses. In our experiments, we used an experimentally defined optimal concentration of poly(I:C) that efficiently and consistently induced SGs in SH-SY5Y cells without overt toxicity (Figure S2A).

We studied mutant FUS recruitment into poly(I:C)-induced SGs in recently generated CRISPR/Cas9 cell lines expressing endogenous FUS lacking NLS (FUSΔNLS) (An et al., 2019) (see STAR Methods). Homozygous FUSΔNLS (ΔNLS_ho) lines and heterozygous FUSΔNLS (ΔNLS_het) lines are characterized by significant and mild cytoplasmic FUS mislocalization, respectively, and endogenous mutant FUS is efficiently recruited into SA-induced SGs (An et al., 2019). We found that diffusely distributed cytoplasmic mutant FUS, but not WT FUS, was readily recruited into poly(I:C)-induced SGs visualized with antibodies against SG marker proteins G3BP1, ATXN2, and YBX1 (Figure 2A; Figure S3A). Poly(I:C)-induced SGs in FUSΔNLS lines were bona fide SGs, because they contained polyadenylated RNA (Figure S3B) and were sensitive to cycloheximide (Figure S3C).

In ΔNLS_het cells, significantly more SGs assembled early after poly(I:C) transfection (2 and 4 h time points), and more SGs were still present 24 h post-transfection compared with WT cells (Figure 2B). In contrast, there was no difference in SG numbers between WT and ΔNLS_het cells during recovery from SA-induced stress (Figure 2B).

We found that upon poly(I:C) stimulation, neuroblastoma cells usually develop larger, few-per-cell SGs, as opposed to multiple small or medium-sized SGs in cells treated with the other stressors tested. We quantified the proportion of cells that possess 1, 2, or >2 SGs per cell and measured the SG area in FUSΔNLS cells subjected to SA for 1.5 h or poly(I:C) for 6 h (these stress durations did not significantly affect the morphology or viability of neuroblastoma cells; Figure S2C). This analysis revealed that a substantial proportion of poly(I:C)-stimulated FUSΔNLS cells contained 1–2 SGs per cell, of larger size, compared with SA-stressed cells, which presented with smaller, more numerous SGs (Figures 2C–2E). Large SGs induced by poly(I:C) were able to sequester almost the entire pool of FUS in ΔNLS_ho lines, leading to its near-complete nuclear depletion (Figure 2F). Finally, poly(I:C) was able to induce FUS-positive SGs in human patient fibroblasts bearing FUS P525L mutation, which were detectable up to 24 h post-transfection (Figure 2G).

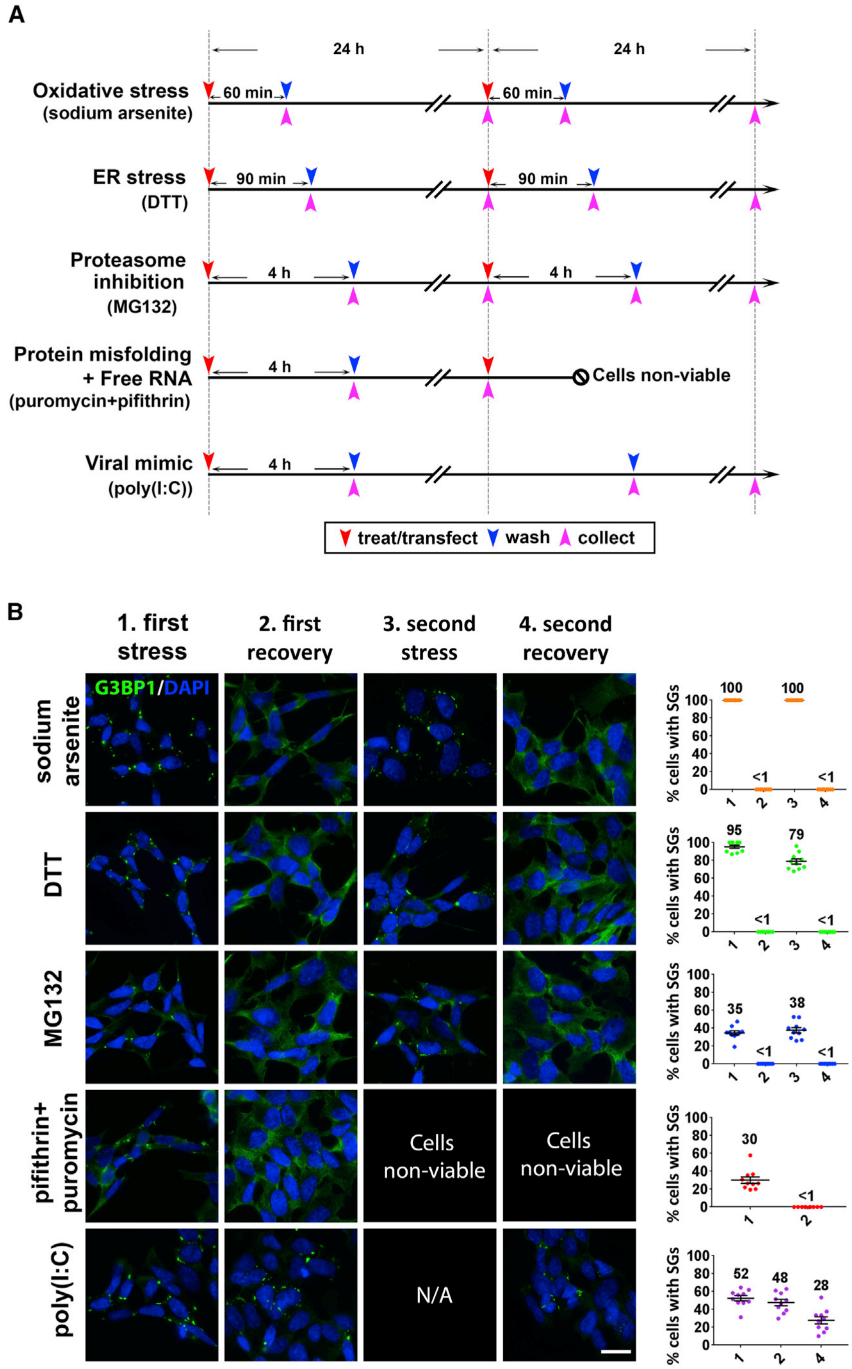

Figure 1. Viral dsRNA Mimic Triggers Formation of Persistent SGs

(A) Timelines for the experiments to study SG persistence in SH-SY5Y cells. Time points for treatments or transfection, wash-off, and cell collection are indicated by red, blue, and pink arrowheads, respectively.

(B) Representative images and quantification of SG-containing cells after stress and recovery. The percentage of SG-positive cells at each time point is indicated on the scatterplots. The x axis labeling (1–4) corresponds to the column labeling (1st stress, 1st recovery, 2nd stress, and 2nd recovery). SGs were visualized using anti-G3BP1 staining. Between 250 and 300 cells were analyzed per time point for each stressor. N/A, not available; single application of poly(I:C) was used. Data are represented as mean ± SEM; mean percentages of cells with SGs are also indicated on the scatterplots. Scale bar, 10 μm.

Thus, mimicking viral infection by dsRNA delivery can cause the prolonged presence of large FUS-positive SGs in cells expressing endogenous mutant FUS.

Mimicking Viral Infection Promotes Formation of Cytoplasmic FUS aggregates

Previously, we and others showed that exogenously expressed mutant FUS forms spontaneous cytoplasmic granules in a fraction of unstressed cells, which we called FUS granules (FGs) (Kino et al., 2011; Shelkovnikova et al., 2014a). Consistently, in some FUSΔNLS lines, the endogenous level of mutant FUS was sufficient to support the assembly of FGs (lines ΔNLS2_het and ΔNLS11_het; Figure 3A). Similar to FGs formed by overexpressed (exogenous) protein (exoFGs), FGs composed of endogenous protein (endoFGs) were negative for core SG proteins G3BP1 and TIAR (Figure 3B) and were sensitive to actinomycin D treatment (Figure S4).

In SA-stressed cells, exoFGs can coalesce into larger assemblies, called FUS aggregates (FAs), which recruit SG proteins (Shelkovnikova et al., 2014a). We found that poly(I:C) was also capable of triggering the formation of FAs in cells with endoFGs (endoFAs) (Figure 3C). These endoFAs were readily distinguishable from FUS-positive SGs because of their grainy, irregularly shaped appearance compared with the amorphous, smooth-edged SGs formed in endoFG-negative FUSΔNLS lines (Figure 3C). endoFAs were able to sequester G3BP1 and TIAR proteins; however, in contrast to FUS-positive SGs, the G3BP1/TIAR signal was intermingled with the FUS signal, forming a patchy pattern within these structures (Figure 3C). Similar to FUS-containing SGs, endoFAs persisted in FUSΔNLS lines and were still detectable in 45.6% ± 2.1% of cells 24 h post-transfection (Figure 3D).

To characterize the dynamics of poly(I:C)-induced FA assembly, we used confocal live imaging of cells with exoFGs formed by GFP-tagged FUS bearing a R522G mutation. Poly(I:C) induced rapid assembly of exoFAs, which grew by

OPEN ACCESS
CellPress

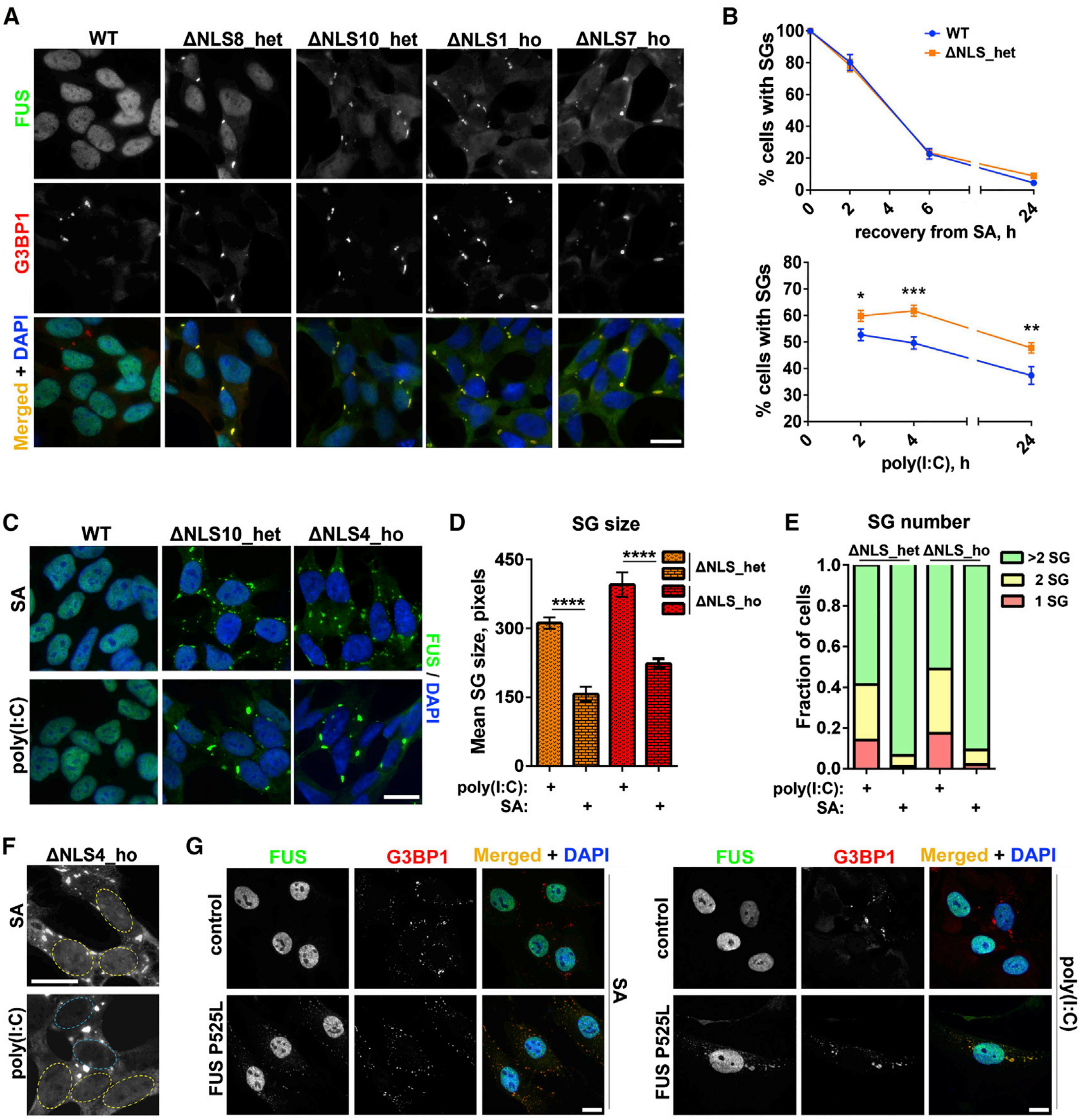

Figure 2. Mutant FUS Is Recruited to poly(I:C)-Induced SGs

(A) Endogenous mutant FUS is highly enriched in poly(I:C)-induced SGs in FUSΔNLS lines. Cells were analyzed 4 h after poly(I:C) transfection. Representative images for two heterozygous FUSΔNLS (ΔNLS_het) lines and two homozygous FUSΔNLS (ΔNLS_ho) lines are shown.

(B) Poly(I:C), but not sodium arsenite (SA), causes persistent SGs in mutant FUS-expressing cells. Quantification of cells containing SGs in ΔNLS_het lines (two lines combined) and WT cells over a 24 h follow-up period post-transfection or post-treatment is shown. Cells were treated with SA for 1 h, washed, and analyzed during recovery, with ≥500 cells analyzed per time point for each line. *$p < 0.05$, **$p < 0.01$, ***$p < 0.001$ (Student's t test).

(C–E) poly(I:C) induces a few large SGs per cell in FUSΔNLS lines. Representative images (C), quantification of the SG area (D), and the fraction of cells containing 1, 2, and >2 SGs (E) in FUSΔNLS cells are shown. In (D) and (E), data for two ΔNLS_het and two ΔNLS_ho lines were combined. Between 250 and 400 cells were analyzed in (D), and between 92 and 141 cells were analyzed in (E). ****$p < 0.0001$ (Student's t test).

(F) Near-complete clearance of mutant FUS from the nucleus in poly(I:C)-stimulated, but not SA-treated, ΔNLS_ho cells. Nuclei are circled. Note the loss of nuclear FUS in two SG-containing cells in the poly(I:C)-stimulated culture (nuclei circled in blue).

(G) poly(I:C) induces FUS-positive SGs in human patient fibroblasts bearing P525L mutation. Fibroblasts were analyzed 24 h post-transfection. Fibroblasts treated with SA for 1 h are shown for comparison.

In (C)–(F), cells were analyzed 6 h after poly(I:C) transfection and 1.5 h after SA addition. In (B) and (D), data are represented as mean ± SEM. Scale bars, 10 μm.

clustering, eventually forming one or two large aggregates per cell, and such cells remained alive for at least 12 h (Figure 3E; Video S1).

To summarize, our data suggest that the presence of endogenous mutant FUS in the cytoplasm is sufficient to form spontaneous FGs, which can become seeds for larger assemblies, the FAs. Mimicking viral infection promotes the formation of FAs composed of such FGs, which can persist in cultured cells for hours and even days.

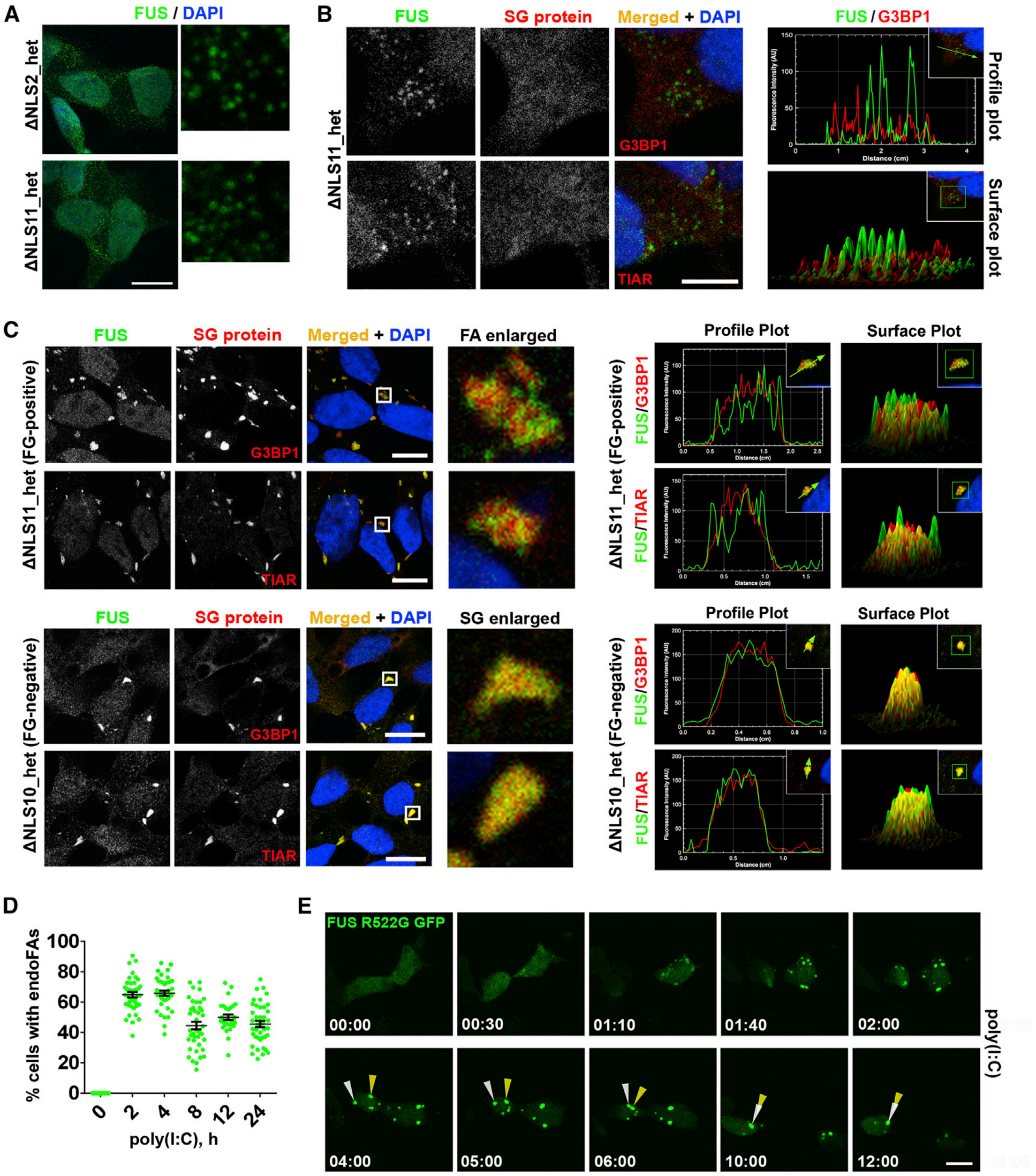

Figure 3. Mimicking Viral Infection Promotes Formation of Cytoplasmic FAs

(A) Spontaneous FGs composed of endogenous protein (endoFGs) are present in the cytoplasm of two ΔNLS_het lines under basal conditions.

(B) SG markers G3BP1 and TIAR are absent from endoFGs.

(C) Non-overlapping localization of mutant FUS and SG proteins in endogenous mutant FUS (endoFUS) aggregates (endoFAs) formed in a FG-positive cell line (ΔNLS11_het) after poly(I:C) transfection. Images of FUS-positive SGs formed in a FG-negative line (ΔNLS10_het) are shown for comparison. Cells were analyzed 4 h post-transfection.

(D) Quantification of the number of cells containing endoFAs in ΔNLS_het lines (data for two lines combined) over a 24-h period post-transfection, where $\geq$500 cells were analyzed per line/time point. Data are represented as mean ± SEM.

(E) Formation of exoFAs in response to poly(I:C). WT cells were transfected with a FUS R522G GFP expression vector; 24 h later, cells were transfected with poly(I:C); and two cells with preformed exoFGs were followed up for 12 h using time-lapse confocal imaging. Two aggregates that eventually fuse to form one large aggregate are indicated with arrowheads. Also see Video S1.

In (A)–(C), representative confocal images (single optical section) are shown. Scale bars, 10 μm.

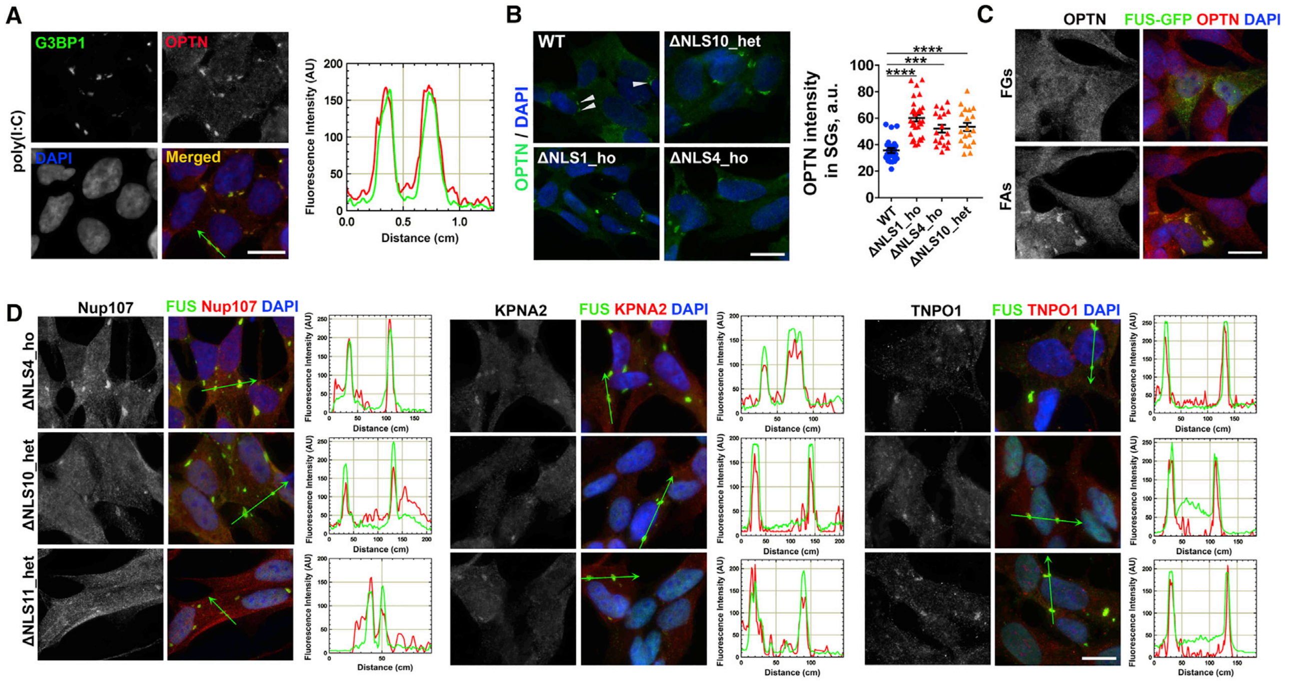

Figure 4. Optineurin and Nucleocytoplasmic Transport Factors Are Recruited into Mutant FUS Assemblies

(A) Optineurin is a component of poly(I:C)-induced SGs in WT cells. Cells were analyzed 6 h post-transfection.

(B) More optineurin is retained in poly(I:C)-induced SGs in FUSΔNLS lines compared with WT cells. Arrowheads indicate optineurin-positive SGs in WT cells. Cells were analyzed 24 h post-transfection. Graph shows optineurin staining intensity in SGs ($n \geq 30$). ***$p < 0.001$, ****$p < 0.0001$ (one-way ANOVA with Dunnett's test). Data are represented as mean ± SEM.

(C) FAs formed by overexpressed GFP-tagged FUS R522G sequester optineurin under basal conditions.

(D) Nucleocytoplasmic transport factors Nup107, KPNA2, and TNPO1 are recruited into mutant FUS cytoplasmic assemblies in poly(I:C)-stimulated FUSΔNLS cells. The ΔNLS11_het cell line contains endoFGs and therefore forms FAs but not SGs. Cells were analyzed 6 h post-transfection.

Scale bars, 10 μm.

Poly(I:C)-Induced Mutant FUS Assemblies Sequester Nucleocytoplasmic Transport Factors and the Autophagy Receptor Optineurin

Aggregates composed of mutant FUS have been found to sequester other proteins, such as survival motor neuron (SMN) complex factors, processing body (P-body), and paraspeckle components, presumably leading to their loss of function (Groen et al., 2013; Shelkovnikova et al., 2014a, 2014b). Optineurin is the autophagy receptor involved in aggrephagy and encoded by an ALS-linked gene, *OPTN*; it regulates critical processes at the crossroads of autophagy and viral infection (Ryan and Tumbarello, 2018). Previously, optineurin was identified as a component of FUS inclusions in ALS-FUS post-mortem tissue (Ito et al., 2011); however, its possible recruitment into mutant FUS assemblies in cell models has not been studied. We examined optineurin distribution in SA- and poly(I:C)-stressed WT neuroblastoma cells and found that this protein was sequestered into both types of SGs (Figure 4A; Figure S5A). Focusing on poly(I:C)-induced SGs, we found that in FUSΔNLS lines, SGs recruited significantly more optineurin compared with WT cells (Figure 4B). Optineurin was also detected in exoFAs formed under basal conditions (Figure 4C). In contrast, another ALS-linked protein and important optineurin interactor, TBK1 (Freischmidt et al., 2015; Wild et al., 2011), was not recruited into FUS-containing SGs or into FAs (Figure S5B). Similarly, the principal component of autophagy-initiating complexes, ULK1, was not detected in SGs (Figure S5B). Thus, abnormal optineurin retention in FUS assemblies would sequester it from autophagic complexes, which may negatively affect macroautophagy.

Disrupted nucleocytoplasmic transport has been implicated in multiple ALS subtypes (Boeynaems et al., 2016), whereas SGs have been reported to sequester nucleocytoplasmic transport factors, including Transportin 1 (TNPO1), the main import receptor for FUS (Zhang et al., 2018). We examined whether poly(I:C)-induced mutant FUS assemblies in FUSΔNLS cells contain the nucleocytoplasmic transport factors TNPO1, karyopherin alpha 2 (KPNA2), and nucleoporins Nup98 and Nup107 (localized in the inner ring and outer ring of the nuclear pore complex [NPC], respectively). We found that TNPO1, KPNA2, and Nup107, but not Nup98, accumulated within poly(I:C)-induced SGs and endoFAs (Figure 4D; Figure S5C).

These data indicate that dsRNA-induced mutant FUS assemblies can cause partial depletion of optineurin and nucleocytoplasmic transport factors.

Mutant FUS-Expressing Cells Are Hypersensitive to dsRNA Toxicity

We next asked whether the presence of mutant FUS may result in increased sensitivity to dsRNA toxicity. The number of apoptotic cells, as visualized by staining for cleaved caspase-3

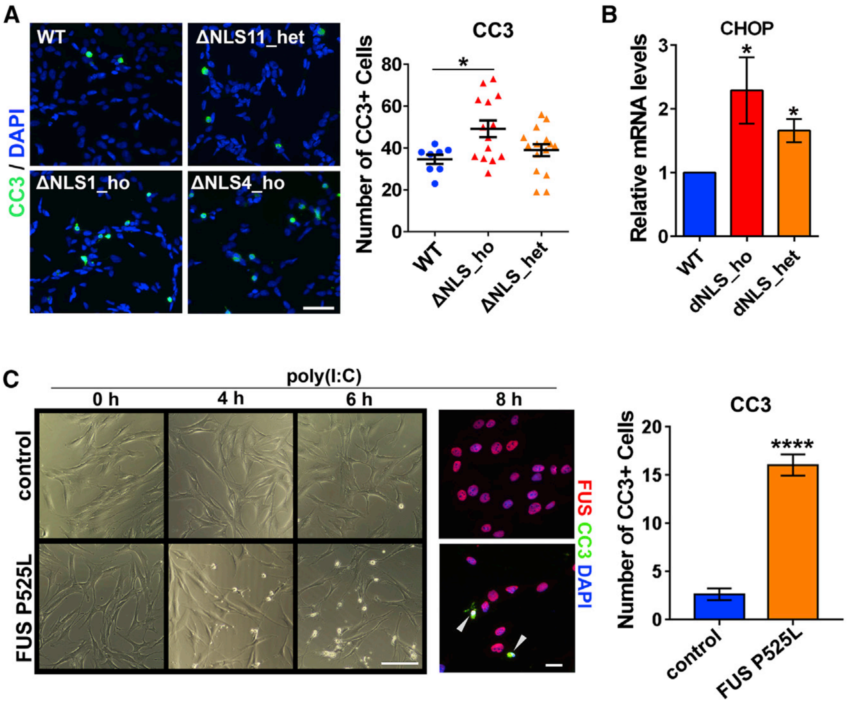

Figure 5. Cells Expressing Mutant FUS Are Hypersensitive to dsRNA Toxicity

(A) Increased number of cleaved CC3-positive cells in poly(I:C)-stimulated ΔNLS_ho cultures. Cells were analyzed 24 h post-transfection. Data for two ΔNLS_het lines and two ΔNLS_ho lines were combined for the graph. 7 fields of view (×20 magnification) were included in the analysis per line. $^{*}p < 0.05$ (one-way ANOVA with Dunnett's test). Representative images are also shown. Scale bar, 50 μm.

(B) Elevated levels of the proapoptotic factor CHOP in FUSΔNLS lines after poly(I:C) stimulation. Cells were analyzed by qRT-PCR 24 h post-transfection. Combined data for three ΔNLS_het lines and three ΔNLS_ho lines are shown (n = 3 for each line). $^{*}p < 0.05$ (Mann-Whitney U test).

(C) Mutant FUS P525L fibroblasts are more sensitive to poly(I:C) compared with control fibroblasts. Representative images and quantification of CC3-positive cells 8 h post-transfection are shown. 9 and 12 fields (×20 magnification) were included into analysis for control and P525L fibroblasts, respectively. Arrowheads indicate CC3-positive cells. $^{****}p < 0.0001$ (Student's t test). Scale bars, 50 and 10 μm for bright-field images and fluorescent images, respectively.

In all panels, data are represented as mean ± SEM.

(CC3), was significantly increased in ΔNLS_ho lines compared with WT cells stimulated with poly(I:C) for 24 h (Figure 5A). FUS-ΔNLS lines also had elevated mRNA levels for the pro-apoptotic factor CHOP (Figure 5B). Furthermore, human patient fibroblasts bearing FUS P525L mutation had dramatically increased susceptibility to poly(I:C)-induced cell death (Figure 5C). Differences in survival between control and patient fibroblasts were already apparent 4 h after poly(I:C) transfection, and quantification of CC3-positive cells revealed that significantly more cells were undergoing apoptosis in mutant fibroblast cultures 8 h post-transfection (Figure 5C).

We concluded that cells expressing mutant FUS are less competent at handling dsRNA-induced toxicity than WT cells.

Type I Interferon Stimulates Accumulation of Normal and Mutant FUS Protein

In FUS proteinopathy, FUS protein accumulates in the cytoplasm in large quantities, and its increased local concentration likely contributes to its aggregation and inclusion formation. Because FUS was previously identified as a potent negative regulator of antiviral gene expression (Amit et al., 2009), we hypothesized that during antiviral response, cells may develop increased demand for FUS protein, leading to its upregulation that may contribute to FUS proteinopathy development. In line with this prediction, qPCR analysis revealed upregulation of FUS mRNA in poly(I:C)-stimulated WT cells (Figure 6A). Type I IFNs are the principal drivers of gene expression changes in response to dsRNA. Thus, we examined whether FUS mRNA upregulation is downstream of IFN signaling. Treatment with IFN-beta, the main type I IFN induced by poly(I:C) in SH-SY5Y cells (Shelkovnikova et al., 2018), increased FUS mRNA levels, with a peak at 4 h followed by a gradual decline (Figure 6B). The increase in FUS mRNA levels was more pronounced in IFN-treated cultures compared with poly(I:C)-stimulated cultures, consistent with induction of IFN response only in a fraction of cells in poly(I:C)-stimulated cultures because of less than 100% transfection efficiency. Consistent with increased mRNA levels, we detected time-dependent accumulation of FUS protein in IFN-treated cells (Figure 6C). FUS protein did not accumulate in poly(I:C)-treated cells despite upregulated mRNA (Figure S6A). This is explained by significant impairment of protein translation in poly(I:C)-transfected cells, but not in IFN-stimulated cells, as confirmed by puromycin incorporation assay (Figure S6B).

FUS mRNA can be upregulated in IFN-treated cells via a transcriptional mechanism or because of its increased stability. We found that FUS pre-mRNA levels in treated cultures did not increase (Figure 6D). Furthermore, FUS mRNA upregulation induced by IFN-beta was still evident in cells upon blocking transcription with actinomycin D or dichlorobenzimidazole riboside 5,6-Dichlorobenzimidazole 1-β-D-ribofuranoside (DRB) (Figure S6C). STAT1 is the main transcriptional mediator of IFN-beta signaling, and although the *FUS* gene possesses a STAT1 binding site in its promoter region, the degree of IFN-induced FUS mRNA upregulation was not prevented by STAT1 knockdown (Figure S6D). Thus, a transcriptional mechanism may not significantly contribute to the effect of IFN-beta on FUS mRNA levels. Because mRNA stability is mainly regulated by polyadenylation, we measured poly(A) (PA) tail length of FUS mRNA by a PCR-based PAT assay. We found that IFN-beta exposure shifted the intensity of the FUS mRNA smear toward longer PA tails (Figure 6E).

We next examined whether IFN-beta exerted a similar effect on mutant FUS. We found that FUS protein levels increased after 24-h IFN-beta treatment not only in WT cells but also in

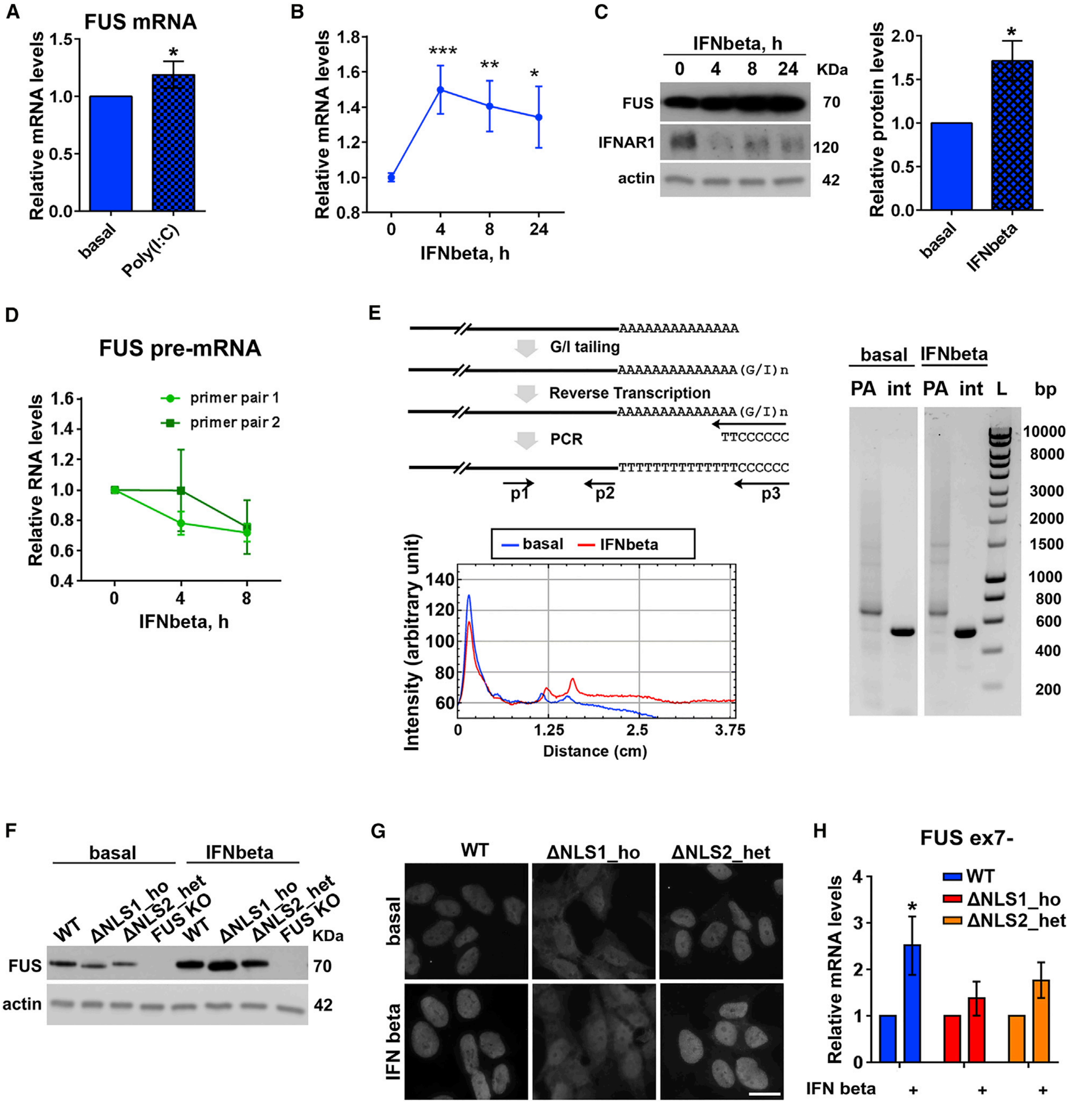

Figure 6. Type I Interferon Promotes Accumulation of Normal and Mutant FUS Protein

(A) FUS mRNA level is increased in poly(I:C)-stimulated WT SH-SY5Y cells 24 h post-transfection as measured by qRT-PCR. n = 4, *$p < 0.05$ (Mann-Whitney U test).

(B) IFN-beta treatment alone upregulates FUS mRNA in WT cells. FUS mRNA levels in IFN-beta-treated cells were measured by qRT-PCR at the indicated time points. n = 4–5. *$p < 0.05$, **$p < 0.01$, ***$p < 0.001$ (Mann-Whitney U test).

(C) IFN-beta treatment causes FUS protein accumulation in WT cells in a time-dependent manner. Representative western blot and quantification of FUS protein levels after 24 h of IFN-beta treatment are shown. Western blot also demonstrates degradation of the IFNAR subunit in IFN-treated cells. n = 3, *$p < 0.05$ (Mann-Whitney U test).

(D) FUS pre-mRNA is not upregulated during IFN-beta treatment. Two pairs of primers mapping to the intron sequences of the *FUS* gene were used for qRT-PCR. n = 3.

(E) FUS mRNA species with longer PATs accumulate in cells treated with IFN-beta as revealed by the PAT assay. The diagram shows the principle of the PAT assay. P1, P2, and P3 are FUS-specific forward, FUS-specific reverse, and universal reverse primers, respectively. PA stands for poly(A) tail (amplified with P1 and P3), and int stands for the internal FUS fragment (amplified with P1 and P2). The electrophoresis image demonstrates a similar band intensity for the internal

(legend continued on next page)

FUSΔNLS lines (Figure 6F). Strikingly, both normal and mutant FUS proteins continued to accumulate 24 h after removing IFN-beta from the culture medium (Figure S6E). At the same time, in IFN-beta-treated cells, subcellular localization of FUS remained unaffected (Figure 6G). IFN treatment alone did not induce SGs in mutant FUS-expressing cells, consistent with previous findings (John and Samuel, 2014) and with a limited effect of IFN on protein translation (Figure S6B). FUS mRNA levels are known to be subject to autoregulation, in which FUS protein binds its own transcript and promotes production of an unstable isoform lacking exon 7; autoregulation ability of mutant FUS is impaired (Zhou et al., 2013). Consistent with defective autoregulation of FUS mutants, FUSΔNLS cells failed to upregulate the exon 7-skipped (ex7−) FUS isoform during IFN treatment (Figure 6H).

Overall, our data indicate that type I IFN, the main component of antiviral signaling, can drive accumulation of mutant FUS protein.

Infection with an RNA Virus Induces FUS Pathology in Mutant FUS-Expressing Cells

To corroborate the data obtained with a viral infection mimic, we next investigated changes in mutant FUS distribution in response to an RNA virus infection. Respiratory syncytial virus (RSV) possesses a negative-sense RNA genome, which gives rise to a dsRNA intermediate in its life cycle; it is capable of maintaining a prolonged stress response accompanied by SG assembly (Groskreutz et al., 2010; Lindquist et al., 2010). Inoculation of WT SH-SY5Y cells with RSV strain A2 led to the appearance of cell clusters with altered cellular morphology, including nuclear swelling and the presence of SGs, 24 h post-infection (Figure 7A). Infected cultures also displayed upregulation of viral infection markers IFN-beta, IFIT3, and CXCL10 (Figure 7B). We next inoculated WT and FUSΔNLS lines and analyzed them 8, 24, and 48 h post-infection. Similar to poly(I:C)-stimulated cells, FG-negative FUSΔNLS lines developed large FUS-positive SGs, whereas a FG-positive line (ΔNLS11_het) developed endoFAs 24 h post-infection (Figure 7C). At this time point, the proportion of SG-positive cells was higher in FUSΔNLS cultures compared with WT cells (Figure 7D). Prolonged RSV infection was toxic for neuroblastoma cells, leading to significant cell death 48 h post-infection, which was more pronounced in FUSΔNLS lines (Figure 7E). FUS-positive SGs were still detectable in some cells at this stage (Figure 7E, insets). RSV infection was also capable of inducing FUS-positive SGs in FUS P525L human patient fibroblasts (Figure 7F). Consistent with poly(I:C) and IFN data (Figure 6), infected WT and FUSΔNLS cells both presented with FUS mRNA upregulation (Figure 7G). Finally, RSV-induced mutant FUS assemblies sequestered optineurin, TNPO1, and Nup107 (Figure 7H).

Overall, core features of FUS pathology triggered by a synthetic dsRNA were observed after infection with a SG-inducing virus.

DISCUSSION

In FUS proteinopathies, cytoplasmic accumulation of FUS can be dramatic, leading to the formation of large inclusions sometimes filling the entire cytosolic space (Deng et al., 2010). Yet even overexpression of ALS-linked FUS mutants in the mammalian CNS is not sufficient to cause overt FUS deposition and proteinopathy. Based on our data, we propose a model whereby a viral infection involving dsRNA response can serve as a trigger, or second hit, for FUS proteinopathy in humans. It does so by causing (cytoplasmic) FUS accumulation, formation of persistent FUS-enriched cytoplasmic assemblies, and impaired aggrephagy. The nature of the third hit, which would facilitate the conversion of dynamic FUS-positive assemblies into stable proteinaceous inclusions, remains unclear. However, one can speculate that viral manipulation of the host RNA levels may be a contributory factor. Viral infections are often associated with attenuated host gene transcription and RNase L-mediated cleavage of cellular RNAs (Abernathy and Glaunsinger, 2015), whereas we and others showed that RNA binding protects FUS from irreversible aggregation (Maharana et al., 2018; Shelkovnikova et al., 2014a). The existence of an additional factor or factors triggering FUS proteinopathy is consistent with the recently proposed multistep model for ALS development, in which intrinsic and extrinsic risk factors interact to realize the genetic predisposition and initiate the pathological process (Al-Chalabi et al., 2014). Considering that ALS is a six-step process and that mutations decrease the number of steps, two or three steps will still be necessary for the development of the disease (Chiò et al., 2018).

Several stresses, including those implicated in neurodegeneration, such as proteasomal dysfunction, oxidative stress, and ER stress, are known to cause formation of FUS-positive assemblies in cultured cells, including neurons (Ederle and Dormann, 2017). However, such assemblies are unstable and readily dissipate when stress is resolved. Moreover, all of these stressors cause an acute response that, if not resolved after a short period (usually up to several hours), kills the cell. In most instances, SGs eventually disassemble to allow restoration of translation, even if the stress persists (Shelkovnikova et al., 2017). SG-inducing viral infections are principally different in this regard, because they result in prolonged and, in some cases, oscillating SG response, which allows phases of active and stalled translation and

FUS fragment but increased intensity of the smear corresponding to the longer PA tails; the intensity profile of the PA tail lanes is also shown. Cells were treated with IFN-beta for 8 h.

(F) Mutant FUS protein accumulates in FUSΔNLS cells upon IFN-beta treatment. Cells were treated with IFN-beta for 24 h. The FUS knockout line was included as a negative control.

(G) IFN-beta treatment does not alter the subcellular localization of normal and mutant FUS. Cells were treated with IFN-beta for 24 h. Scale bar, 10 μm.

(H) Levels of FUS ex7− mRNA transcript significantly increase in WT lines, but not in FUSΔNLS lines, upon IFN-beta exposure. Cells were treated with IFN-beta for 24 h and analyzed by qRT-PCR. n = 4, *$p < 0.05$ (Mann-Whitney U test).

In all panels, data are represented as mean ± SEM.

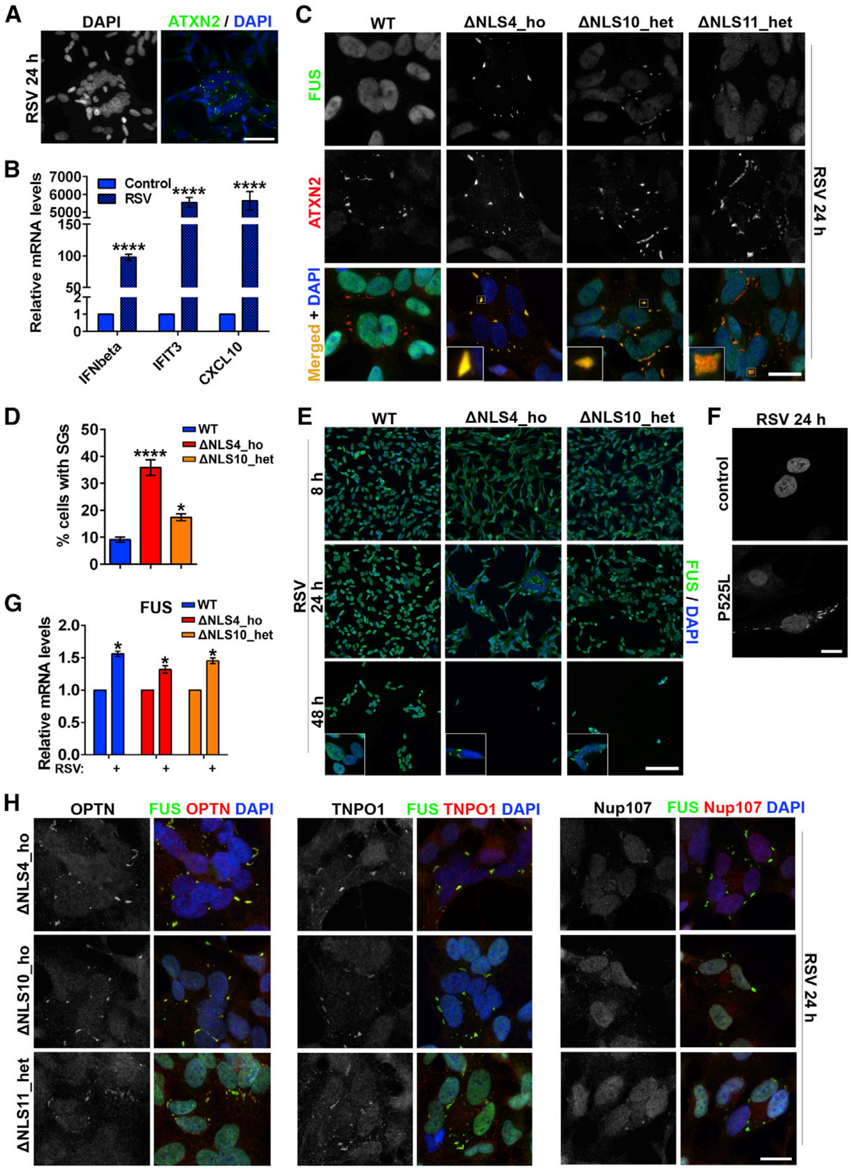

Figure 7. Infection with RSV Induces Cytoplasmic FUS Assemblies and Is Toxic in Mutant FUS-Expressing Cells

(A) RSV infection of SH-SY5Y cells leads to the appearance of clusters of cells with swollen nuclei and cytoplasmic SGs. Representative images of WT cells 24 h post-infection are shown.

(B) Upregulation of antiviral markers in RSV-infected WT cells as analyzed by qRT-PCR. n = 4, ****$p < 0.0001$ (Student's t test).

(C) RSV-inoculated cells develop FUS-positive SGs (ΔNLS4_ho and ΔNLS10_het lines) and FAs (ΔNLS11_het line). Cells were analyzed 24 h post-infection.

(D) More cells develop SGs in FUSΔNLS cultures compared with WT cells upon RSV infection. The proportion of SG-containing cells was quantified 24 h after infection. *$p < 0.05$, ****$p < 0.0001$ (one-way ANOVA with Dunnett's test).

(E) RSV infection is more toxic for FUSΔNLS lines compared with WT cells. Equal numbers of cells were seeded on coverslips. Cells were fixed for analysis at the indicated time points. FUS-positive SGs are still detectable in RSV-infected FUSΔNLS lines 48 h post-infection (insets).

(F) RSV-infected human patient fibroblasts bearing P525L mutation develop FUS-positive SGs 24 h post-inoculation.

(G) FUS mRNA is upregulated in WT and FUSΔNLS cells in response to RSV infection. FUS expression was analyzed by qRT-PCR 24 h post-inoculation. n = 4, *$p < 0.05$ (Student's t test).

(H) Mutant FUS assemblies contain optineurin and nucleocytoplasmic transport factors TNPO1 and Nup107. Cells were fixed and stained 24 h post-inoculation.

In all panels, cells were infected with RSV strain A2 at a multiplicity of infection (MOI) of 10 and analyzed at the indicated time points. In (B), (D), and (G), data are represented as mean ± SEM. In (A), (C), (E), (F), and (H), representative images are shown. Scale bars, (A) 50 μm; (C), (F), and (H) 10 μm; and (E) 100 μm.

ensures cell survival (Ruggieri et al., 2012). Thus, antiviral response may initiate and/or promote FUS proteinopathy in neurons and glia while keeping these cells alive for a period long enough to allow FUS inclusion formation.

Here, we refer to FAs composed of spontaneous FGs and FUS-positive SGs in cultured cells as mutant FUS-containing cytoplasmic assemblies. The ability of mutant FUS to assemble into FGs under basal conditions and subsequently into FAs under conditions of stress in cultured cells, including neurons, has been confirmed by us, as well as by several other groups (Japtok et al., 2015; Kino et al., 2011; Lenzi et al., 2015; Shelkovnikova et al., 2014a). Regardless of the relative contribution of FUS-positive SGs and FAs to the disease pathogenesis, in the current study, we have shown that antiviral signaling can promote the formation and persistence of both types of assemblies.

Compromised autophagic clearance has been heavily implicated in the proteinopathy development in different ALS subtypes (Weishaupt et al., 2016). Previously, mislocalized FUS has been shown to negatively affect autophagic protein clearance, which can contribute to FUS proteinopathy development (Ryu et al., 2014; Soo et al., 2015). Our findings corroborate the histopathological data (Ito et al., 2011) on possible loss of function of optineurin and hence impairment of aggrephagy in ALS-FUS. Autophagy is known to function in the control of viral replication and to exert other antiviral effects; many viruses have evolved mechanisms to inhibit autophagy at different levels (Lee and Iwasaki, 2008). Therefore, a viral infection could exacerbate pre-existing defects in autophagy in mutant FUS-expressing cells.

Available data suggest that even subtle changes in FUS levels can trigger motor neuron pathology. ALS-causative mutations in the *FUS* 3′ UTR, which cause increased protein levels, have been

described (Sabatelli et al., 2013). In the current study, we report the ability of a physiologically relevant molecule, type I IFN, to trigger accumulation of FUS protein. In addition, although IFN per se does not induce SG assembly, it can potentiate SG formation in infected cells (John and Samuel, 2014; Ruggieri et al., 2012). Type I IFN induction is not limited to viral infection and can be caused by other immune stimuli; however, sustained IFN expression is observed only during antiviral signaling (Amit et al., 2009). Both neurons and glia express IFNs and their receptors (Chhatbar et al., 2018). Interestingly, we found that one of the two IFN receptor subunits, IFNAR1, is highly expressed specifically in ventral horn neurons of the spinal cord and that it is depleted from the spinal cord of ALS-FUS patients (Figure S7). Because IFNAR1 undergoes ligand-dependent degradation during viral infection (de Weerd and Nguyen, 2012), this finding is consistent with the idea that sustained antiviral signaling might take place in the nervous system of ALS-FUS patients.

Viral infections are known to promote formation of another type of RNA granule, paraspeckles (Imamura et al., 2014), whereas spinal neurons in ALS, including ALS-FUS, are characterized by paraspeckle hyperassembly (An et al., 2019; Nishimoto et al., 2013; Shelkovnikova et al., 2018). Activation of paraspeckle signaling in the ALS spinal cord provides yet more evidence in support to the hypothesis of activated antiviral response in this disease.

The model proposed here is fully applicable for ALS-FUS, but for FTLD-FUS cases, which usually do not involve FUS mutations, additional factors are required to cause nuclear import defect. However, such a defect might be caused by viral subversion of the NPC. Viruses are known to hijack NPC components and other factors to enable trafficking of viral proteins (Le Sage and Mouland, 2013). Furthermore, some viral proteins have a high affinity to TNPO1. For example, enterovirus and cardiovirus infections alter NPC composition to relocalize some nuclear proteins to the cytoplasm, whereas enteroviruses are known to induce degradation of nucleoporins Nup62, Nup98, and Nup153 (Hindley et al., 2007). A combination of a viral infection and the presence of persistent FUS assemblies that sequester NPC components can have an additive negative effect on nucleocytoplasmic transport.

In addition to the existence of an epidemiological link between viral infections and ALS (Ravits, 2005; Vandenberghe et al., 2010), several viruses are known to cause cellular and molecular phenotypes typical for ALS. For example, enteroviral and encephalomyelitis infections result in cytoplasmic aggregation of TDP-43 *in vivo* in the murine CNS (Masaki et al., 2019; Xue et al., 2018). Enteroviruses (including poliovirus) possess a dsRNA intermediate in their life cycle that triggers SG formation (Lloyd, 2016). Incomplete and non-penetrance is common for FUS mutations, with the age of onset varying from early 20 s to late 70 s even within the same family (Mackenzie et al., 2010). Thus, one can speculate that common viral infections can trigger the disease, which otherwise would not manifest, by dysregulating cellular pathways already perturbed in individuals genetically predisposed to develop ALS.

In conclusion, our study provides a framework for investigating the role of the antiviral signaling in FUS proteinopathies. Further studies are needed to establish whether viral infection would be sufficient to induce formation of FUS inclusions in motor neurons derived from ALS-FUS patient induced pluripotent stem cells (iPSCs) and, most interestingly, in the available rodent models of FUS pathology.

STAR★METHODS

Detailed methods are provided in the online version of this paper and include the following:

- KEY RESOURCES TABLE
- LEAD CONTACT AND MATERIALS AVAILABILITY
- EXPERIMENTAL MODEL AND SUBJECT DETAILS
  - Cell lines
  - Virus strain
  - Human post-mortem tissue
- METHOD DETAILS
  - Cell transfection and treatments
  - Immunocytochemistry, light and confocal microscopy
  - RNA extraction, qRT-PCR and poly(A)-tail (PAT) length assay
  - Western blotting
- QUANTIFICATION AND STATISTICAL ANALYSIS
- DATA AND CODE AVAILABILITY

SUPPLEMENTAL INFORMATION

Supplemental Information can be found online at https://doi.org/10.1016/j.celrep.2019.11.094.

ACKNOWLEDGMENTS

We acknowledge the Sheffield Brain Tissue Bank, London Neurodegenerative Diseases Brain Bank, and Vincenzo La Bella (ALS Clinical Research Center, Palermo University, Italy) for providing human materials. The study was supported by fellowships from Medical Research Foundation and Motor Neurone Disease Association (Shelkovnikova/Oct17/968-799) to T.A.S. and by a Motor Neuron Disease Association research grant to V.L.B. (Buchman/Apr13/6096). H.A. is a recipient of a Cardiff University/China Scholarship Council PhD studentship.

AUTHOR CONTRIBUTIONS

T.A.S. conceived the research; T.A.S., H.A., I.R.H., J.S.T., and V.L.B. designed experiments and analyzed data; H.A., L.S., I.R.H., J.S.T., and T.A.S. performed experiments; H.A., T.A.S., and V.L.B. wrote the manuscript, with input from all authors; and all authors read and approved the final version of the manuscript.

DECLARATION OF INTERESTS

The authors declare no competing interests.

Received: March 1, 2019
Revised: October 16, 2019
Accepted: November 22, 2019
Published: December 24, 2019

REFERENCES

Abernathy, E., and Glaunsinger, B. (2015). Emerging roles for RNA degradation in viral replication and antiviral defense. Virology *479–480*, 600–608.

Al-Chalabi, A., Calvo, A., Chio, A., Colville, S., Ellis, C.M., Hardiman, O., Heverin, M., Howard, R.S., Huisman, M.H.B., Keren, N., et al. (2014). Analysis

of amyotrophic lateral sclerosis as a multistep process: a population-based modelling study. Lancet Neurol. *13*, 1108–1113.

Alfahad, T., and Nath, A. (2013). Retroviruses and amyotrophic lateral sclerosis. Antiviral Res. *99*, 180–187.

Amit, I., Garber, M., Chevrier, N., Leite, A.P., Donner, Y., Eisenhaure, T., Guttman, M., Grenier, J.K., Li, W., Zuk, O., et al. (2009). Unbiased reconstruction of a mammalian transcriptional network mediating pathogen responses. Science *326*, 257–263.

An, H., Skelt, L., Notaro, A., Highley, J.R., Fox, A.H., La Bella, V., Buchman, V.L., and Shelkovnikova, T.A. (2019). ALS-linked FUS mutations confer loss and gain of function in the nucleus by promoting excessive formation of dysfunctional paraspeckles. Acta Neuropathol. Commun. *7*, 7.

Bentmann, E., Haass, C., and Dormann, D. (2013). Stress granules in neurodegeneration–lessons learnt from TAR DNA binding protein of 43 kDa and fused in sarcoma. FEBS J. *280*, 4348–4370.

Boeynaems, S., Bogaert, E., Van Damme, P., and Van Den Bosch, L. (2016). Inside out: the role of nucleocytoplasmic transport in ALS and FTLD. Acta Neuropathol. *132*, 159–173.

Bosco, D.A., Lemay, N., Ko, H.K., Zhou, H., Burke, C., Kwiatkowski, T.J., Jr., Sapp, P., McKenna-Yasek, D., Brown, R.H., Jr., and Hayward, L.J. (2010). Mutant FUS proteins that cause amyotrophic lateral sclerosis incorporate into stress granules. Hum. Mol. Genet. *19*, 4160–4175.

Bounedjah, O., Desforges, B., Wu, T.D., Pioche-Durieu, C., Marco, S., Hamon, L., Curmi, P.A., Guerquin-Kern, J.L., Piétrement, O., and Pastré, D. (2014). Free mRNA in excess upon polysome dissociation is a scaffold for protein multimerization to form stress granules. Nucleic Acids Res. *42*, 8678–8691.

Celeste, D.B., and Miller, M.S. (2018). Reviewing the evidence for viruses as environmental risk factors for ALS: A new perspective. Cytokine *108*, 173–178.

Chhatbar, C., Detje, C.N., Grabski, E., Borst, K., Spanier, J., Ghita, L., Elliott, D.A., Jordão, M.J.C., Mueller, N., Sutton, J., et al. (2018). Type I Interferon Receptor Signaling of Neurons and Astrocytes Regulates Microglia Activation during Viral Encephalitis. Cell Rep *25*, 118–129.

Chiò, A., Restagno, G., Brunetti, M., Ossola, I., Calvo, A., Mora, G., Sabatelli, M., Monsurrò, M.R., Battistini, S., Mandrioli, J., et al.; ITALSGEN Consortium (2009). Two Italian kindreds with familial amyotrophic lateral sclerosis due to FUS mutation. Neurobiol. Aging *30*, 1272–1275.

Chiò, A., Mazzini, L., D'Alfonso, S., Corrado, L., Canosa, A., Moglia, C., Manera, U., Bersano, E., Brunetti, M., Barberis, M., et al. (2018). The multistep hypothesis of ALS revisited: The role of genetic mutations. Neurology *91*, e635–e642.

de Weerd, N.A., and Nguyen, T. (2012). The interferons and their receptors–distribution and regulation. Immunol. Cell Biol. *90*, 483–491.

Deng, H.X., Zhai, H., Bigio, E.H., Yan, J., Fecto, F., Ajroud, K., Mishra, M., Ajroud-Driss, S., Heller, S., Sufit, R., et al. (2010). FUS-immunoreactive inclusions are a common feature in sporadic and non-SOD1 familial amyotrophic lateral sclerosis. Ann. Neurol. *67*, 739–748.

Deng, H., Gao, K., and Jankovic, J. (2014). The role of FUS gene variants in neurodegenerative diseases. Nat. Rev. Neurol. *10*, 337–348.

Devoy, A., Kalmar, B., Stewart, M., Park, H., Burke, B., Noy, S.J., Redhead, Y., Humphrey, J., Lo, K., Jaeger, J., et al. (2017). Humanized mutant FUS drives progressive motor neuron degeneration without aggregation in 'FUSDelta14' knockin mice. Brain *140*, 2797–2805.

Dormann, D., Rodde, R., Edbauer, D., Bentmann, E., Fischer, I., Hruscha, A., Than, M.E., Mackenzie, I.R., Capell, A., Schmid, B., et al. (2010). ALS-associated fused in sarcoma (FUS) mutations disrupt Transportin-mediated nuclear import. EMBO J. *29*, 2841–2857.

Ederle, H., and Dormann, D. (2017). TDP-43 and FUS en route from the nucleus to the cytoplasm. FEBS Lett. *591*, 1489–1507.

Freischmidt, A., Wieland, T., Richter, B., Ruf, W., Schaeffer, V., Müller, K., Marroquin, N., Nordin, F., Hübers, A., Weydt, P., et al. (2015). Haploinsufficiency of TBK1 causes familial ALS and fronto-temporal dementia. Nat. Neurosci. *18*, 631–636.

Groen, E.J., Fumoto, K., Blokhuis, A.M., Engelen-Lee, J., Zhou, Y., van den Heuvel, D.M., Koppers, M., van Diggelen, F., van Heest, J., Demmers, J.A., et al. (2013). ALS-associated mutations in FUS disrupt the axonal distribution and function of SMN. Hum. Mol. Genet. *22*, 3690–3704.

Groskreutz, D.J., Babor, E.C., Monick, M.M., Varga, S.M., and Hunninghake, G.W. (2010). Respiratory syncytial virus limits alpha subunit of eukaryotic translation initiation factor 2 (eIF2alpha) phosphorylation to maintain translation and viral replication. J. Biol. Chem. *285*, 24023–24031.

Hindley, C.E., Lawrence, F.J., and Matthews, D.A. (2007). A role for transportin in the nuclear import of adenovirus core proteins and DNA. Traffic *8*, 1313–1322.

Huang, C., Zhou, H., Tong, J., Chen, H., Liu, Y.J., Wang, D., Wei, X., and Xia, X.G. (2011). FUS transgenic rats develop the phenotypes of amyotrophic lateral sclerosis and frontotemporal lobar degeneration. PLoS Genet. *7*, e1002011.

Imamura, K., Imamachi, N., Akizuki, G., Kumakura, M., Kawaguchi, A., Nagata, K., Kato, A., Kawaguchi, Y., Sato, H., Yoneda, M., et al. (2014). Long noncoding RNA NEAT1-dependent SFPQ relocation from promoter region to paraspeckle mediates IL8 expression upon immune stimuli. Mol. Cell *53*, 393–406.

Ito, H., Fujita, K., Nakamura, M., Wate, R., Kaneko, S., Sasaki, S., Yamane, K., Suzuki, N., Aoki, M., Shibata, N., et al. (2011). Optineurin is co-localized with FUS in basophilic inclusions of ALS with FUS mutation and in basophilic inclusion body disease. Acta Neuropathol. *121*, 555–557.

Japtok, J., Lojewski, X., Naumann, M., Klingenstein, M., Reinhardt, P., Sterneckert, J., Putz, S., Demestre, M., Boeckers, T.M., Ludolph, A.C., et al. (2015). Stepwise acquirement of hallmark neuropathology in FUS-ALS iPSC models depends on mutation type and neuronal aging. Neurobiol. Dis. *82*, 420–429.

John, L., and Samuel, C.E. (2014). Induction of stress granules by interferon and down-regulation by the cellular RNA adenosine deaminase ADAR1. Virology *454–455*, 299–310.

Kino, Y., Washizu, C., Aquilanti, E., Okuno, M., Kurosawa, M., Yamada, M., Doi, H., and Nukina, N. (2011). Intracellular localization and splicing regulation of FUS/TLS are variably affected by amyotrophic lateral sclerosis-linked mutations. Nucleic Acids Res. *39*, 2781–2798.

Kwiatkowski, T.J., Jr., Bosco, D.A., Leclerc, A.L., Tamrazian, E., Vanderburg, C.R., Russ, C., Davis, A., Gilchrist, J., Kasarskis, E.J., Munsat, T., et al. (2009). Mutations in the FUS/TLS gene on chromosome 16 cause familial amyotrophic lateral sclerosis. Science *323*, 1205–1208.

Le Sage, V., and Mouland, A.J. (2013). Viral subversion of the nuclear pore complex. Viruses *5*, 2019–2042.

Lee, H.K., and Iwasaki, A. (2008). Autophagy and antiviral immunity. Curr. Opin. Immunol. *20*, 23–29.

Lenzi, J., De Santis, R., de Turris, V., Morlando, M., Laneve, P., Calvo, A., Caliendo, V., Chiò, A., Rosa, A., and Bozzoni, I. (2015). ALS mutant FUS proteins are recruited into stress granules in induced pluripotent stem cell-derived motoneurons. Dis. Model. Mech. *8*, 755–766.

Li, Y.R., King, O.D., Shorter, J., and Gitler, A.D. (2013). Stress granules as crucibles of ALS pathogenesis. J. Cell Biol. *201*, 361–372.

Lindquist, M.E., Lifland, A.W., Utley, T.J., Santangelo, P.J., and Crowe, J.E., Jr. (2010). Respiratory syncytial virus induces host RNA stress granules to facilitate viral replication. J. Virol. *84*, 12274–12284.

Lloyd, R.E. (2016). Enterovirus Control of Translation and RNA Granule Stress Responses. Viruses *8*, 93.

Lo Bello, M., Di Fini, F., Notaro, A., Spataro, R., Conforti, F.L., and La Bella, V. (2017). ALS-Related Mutant FUS Protein Is Mislocalized to Cytoplasm and Is Recruited into Stress Granules of Fibroblasts from Asymptomatic FUS P525L Mutation Carriers. Neurodegener. Dis. *17*, 292–303.

López-Erauskin, J., Tadokoro, T., Baughn, M.W., Myers, B., McAlonis-Downes, M., Chillon-Marinas, C., Asiaban, J.N., Artates, J., Bui, A.T., Vetto, A.P., et al. (2018). ALS/FTD-Linked Mutation in FUS Suppresses Intra-axonal Protein Synthesis and Drives Disease Without Nuclear Loss-of-Function of FUS. Neuron *100*, 816–830.e7.

Mackenzie, I.R.A., Rademakers, R., and Neumann, M. (2010). TDP-43 and FUS in amyotrophic lateral sclerosis and frontotemporal dementia. Lancet Neurol. *9*, 995–1007.

Maharana, S., Wang, J., Papadopoulos, D.K., Richter, D., Pozniakovsky, A., Poser, I., Bickle, M., Rizk, S., Guillén-Boixet, J., Franzmann, T.M., et al. (2018). RNA buffers the phase separation behavior of prion-like RNA binding proteins. Science *360*, 918–921.

Martyn, C.N., Barker, D.J., and Osmond, C. (1988). Motoneuron disease and past poliomyelitis in England and Wales. Lancet *1*, 1319–1322.

Masaki, K., Sonobe, Y., Ghadge, G., Pytel, P., and Roos, R.P. (2019). TDP-43 proteinopathy in Theiler's murine encephalomyelitis virus infection. PLoS Pathog. *15*, e1007574.

Mateju, D., Franzmann, T.M., Patel, A., Kopach, A., Boczek, E.E., Maharana, S., Lee, H.O., Carra, S., Hyman, A.A., and Alberti, S. (2017). An aberrant phase transition of stress granules triggered by misfolded protein and prevented by chaperone function. EMBO J. *36*, 1669–1687.

McCormick, C., and Khaperskyy, D.A. (2017). Translation inhibition and stress granules in the antiviral immune response. Nat. Rev. Immunol. *17*, 647–660.

Neumann, M., Rademakers, R., Roeber, S., Baker, M., Kretzschmar, H.A., and Mackenzie, I.R. (2009). A new subtype of frontotemporal lobar degeneration with FUS pathology. Brain *132*, 2922–2931.

Nishimoto, Y., Nakagawa, S., Hirose, T., Okano, H.J., Takao, M., Shibata, S., Suyama, S., Kuwako, K., Imai, T., Murayama, S., et al. (2013). The long non-coding RNA nuclear-enriched abundant transcript 1_2 induces paraspeckle formation in the motor neuron during the early phase of amyotrophic lateral sclerosis. Mol. Brain *6*, 31.

Peters, O.M., Ghasemi, M., and Brown, R.H., Jr. (2015). Emerging mechanisms of molecular pathology in ALS. J. Clin. Invest. *125*, 2548.

Ratti, A., and Buratti, E. (2016). Physiological functions and pathobiology of TDP-43 and FUS/TLS proteins. J. Neurochem. *138* (*Suppl 1*), 95–111.

Ravits, J. (2005). Sporadic amyotrophic lateral sclerosis: a hypothesis of persistent (non-lytic) enteroviral infection. Amyotroph. Lateral Scler. Other Motor Neuron Disord. *6*, 77–87.

Robinson, H.K., Deykin, A.V., Bronovitsky, E.V., Ovchinnikov, R.K., Ustyugov, A.A., Shelkovnikova, T.A., Kukharsky, M.S., Ermolkevich, T.G., Goldman, I.L., Sadchikova, E.R., et al. (2015). Early lethality and neuronal proteinopathy in mice expressing cytoplasm-targeted FUS that lacks the RNA recognition motif. Amyotroph. Lateral Scler. Frontotemporal Degener. *16*, 402–409.

Ruggieri, A., Dazert, E., Metz, P., Hofmann, S., Bergeest, J.P., Mazur, J., Bankhead, P., Hiet, M.S., Kallis, S., Alvisi, G., et al. (2012). Dynamic oscillation of translation and stress granule formation mark the cellular response to virus infection. Cell Host Microbe *12*, 71–85.

Ryan, T.A., and Tumbarello, D.A. (2018). Optineurin: A Coordinator of Membrane-Associated Cargo Trafficking and Autophagy. Front. Immunol. *9*, 1024.

Ryu, H.H., Jun, M.H., Min, K.J., Jang, D.J., Lee, Y.S., Kim, H.K., and Lee, J.A. (2014). Autophagy regulates amyotrophic lateral sclerosis-linked fused in sarcoma-positive stress granules in neurons. Neurobiol. Aging *35*, 2822–2831.

Sabatelli, M., Moncada, A., Conte, A., Lattante, S., Marangi, G., Luigetti, M., Lucchini, M., Mirabella, M., Romano, A., Del Grande, A., et al. (2013). Mutations in the 3′ untranslated region of FUS causing FUS overexpression are associated with amyotrophic lateral sclerosis. Hum. Mol. Genet. *22*, 4748–4755.

Sama, R.R., Ward, C.L., Kaushansky, L.J., Lemay, N., Ishigaki, S., Urano, F., and Bosco, D.A. (2013). FUS/TLS assembles into stress granules and is a prosurvival factor during hyperosmolar stress. J. Cell. Physiol. *228*, 2222–2231.

Scekic-Zahirovic, J., Sendscheid, O., El Oussini, H., Jambeau, M., Sun, Y., Mersmann, S., Wagner, M., Dieterlé, S., Sinniger, J., Dirrig-Grosch, S., et al. (2016). Toxic gain of function from mutant FUS protein is crucial to trigger cell autonomous motor neuron loss. EMBO J. *35*, 1077–1097.

Schneider, C.A., Rasband, W.S., and Eliceiri, K.W. (2012). NIH Image to ImageJ: 25 years of image analysis. Nat. Methods *9*, 671–675.

Sharma, A., Lyashchenko, A.K., Lu, L., Nasrabady, S.E., Elmaleh, M., Mendelsohn, M., Nemes, A., Tapia, J.C., Mentis, G.Z., and Shneider, N.A. (2016). ALS-associated mutant FUS induces selective motor neuron degeneration through toxic gain of function. Nat. Commun. *7*, 10465.

Shelkovnikova, T.A., Peters, O.M., Deykin, A.V., Connor-Robson, N., Robinson, H., Ustyugov, A.A., Bachurin, S.O., Ermolkevich, T.G., Goldman, I.L., Sadchikova, E.R., et al. (2013a). Fused in sarcoma (FUS) protein lacking nuclear localization signal (NLS) and major RNA binding motifs triggers proteinopathy and severe motor phenotype in transgenic mice. J. Biol. Chem. *288*, 25266–25274.

Shelkovnikova, T.A., Robinson, H.K., Connor-Robson, N., and Buchman, V.L. (2013b). Recruitment into stress granules prevents irreversible aggregation of FUS protein mislocalized to the cytoplasm. Cell Cycle *12*, 3194–3202.

Shelkovnikova, T.A., Robinson, H.K., Southcombe, J.A., Ninkina, N., and Buchman, V.L. (2014a). Multistep process of FUS aggregation in the cell cytoplasm involves RNA-dependent and RNA-independent mechanisms. Hum. Mol. Genet. *23*, 5211–5226.

Shelkovnikova, T.A., Robinson, H.K., Troakes, C., Ninkina, N., and Buchman, V.L. (2014b). Compromised paraspeckle formation as a pathogenic factor in FUSopathies. Hum. Mol. Genet. *23*, 2298–2312.

Shelkovnikova, T.A., Dimasi, P., Kukharsky, M.S., An, H., Quintiero, A., Schirmer, C., Buée, L., Galas, M.C., and Buchman, V.L. (2017). Chronically stressed or stress-preconditioned neurons fail to maintain stress granule assembly. Cell Death Dis. *8*, e2788.

Shelkovnikova, T.A., Kukharsky, M.S., An, H., Dimasi, P., Alexeeva, S., Shabir, O., Heath, P.R., and Buchman, V.L. (2018). Protective paraspeckle hyper-assembly downstream of TDP-43 loss of function in amyotrophic lateral sclerosis. Mol. Neurodegener. *13*, 30.

Soo, K.Y., Sultana, J., King, A.E., Atkinson, R., Warraich, S.T., Sundaramoorthy, V., Blair, I., Farg, M.A., and Atkin, J.D. (2015). ALS-associated mutant FUS inhibits macroautophagy which is restored by overexpression of Rab1. Cell Death Discov. *1*, 15030.

Vance, C., Rogelj, B., Hortobágyi, T., De Vos, K.J., Nishimura, A.L., Sreedharan, J., Hu, X., Smith, B., Ruddy, D., Wright, P., et al. (2009). Mutations in FUS, an RNA processing protein, cause familial amyotrophic lateral sclerosis type 6. Science *323*, 1208–1211.

Vandenberghe, N., Leveque, N., Corcia, P., Brunaud-Danel, V., Salort-Campana, E., Besson, G., Tranchant, C., Clavelou, P., Beaulieux, F., Ecochard, R., et al. (2010). Cerebrospinal fluid detection of enterovirus genome in ALS: a study of 242 patients and 354 controls. Amyotroph. Lateral Scler. *11*, 277–282.

Weishaupt, J.H., Hyman, T., and Dikic, I. (2016). Common Molecular Pathways in Amyotrophic Lateral Sclerosis and Frontotemporal Dementia. Trends Mol. Med. *22*, 769–783.

Weissbach, R., and Scadden, A.D. (2012). Tudor-SN and ADAR1 are components of cytoplasmic stress granules. RNA *18*, 462–471.

White, J.P., and Lloyd, R.E. (2012). Regulation of stress granules in virus systems. Trends Microbiol. *20*, 175–183.

Wild, P., Farhan, H., McEwan, D.G., Wagner, S., Rogov, V.V., Brady, N.R., Richter, B., Korac, J., Waidmann, O., Choudhary, C., et al. (2011). Phosphorylation of the autophagy receptor optineurin restricts Salmonella growth. Science *333*, 228–233.

Wolozin, B. (2012). Regulated protein aggregation: stress granules and neurodegeneration. Mol. Neurodegener. *7*, 56.

Xue, Y.C., Ruller, C.M., Fung, G., Mohamud, Y., Deng, H., Liu, H., Zhang, J., Feuer, R., and Luo, H. (2018). Enteroviral Infection Leads to Transactive Response DNA-Binding Protein 43 Pathology *in Vivo*. Am. J. Pathol. *188*, 2853–2862.

Zhang, K., Daigle, J.G., Cunningham, K.M., Coyne, A.N., Ruan, K., Grima, J.C., Bowen, K.E., Wadhwa, H., Yang, P., Rigo, F., et al. (2018). Stress Granule Assembly Disrupts Nucleocytoplasmic Transport. Cell *173*, 958–971.

Zhou, Y., Liu, S., Liu, G., Oztürk, A., and Hicks, G.G. (2013). ALS-associated FUS mutations result in compromised FUS alternative splicing and autoregulation. PLoS Genet. *9*, e1003895.

STAR★METHODS

KEY RESOURCES TABLE

REAGENT or RESOURCE	SOURCE	IDENTIFIER
Antibodies		
Rabbit monoclonal anti-eIF2α (phosphorylated, Ser51)	Abcam	Cat#ab32157; RRID:AB_732117
Rabbit polyclonal anti-ULK1	Abcam	Cat#ab240916
Mouse monoclonal anti-G3BP1	BD Biosciences	Cat#611126; RRID:AB_398437
Mouse monoclonal anti-TIAR	BD Biosciences	Cat#610352; RRID:AB_397742
Rabbit polyclonal anti-IFNAR1	Bethyl	Cat#A304-290A; RRID:AB_2620486
Rabbit polyclonal anti-OPTN	Bethyl	Cat#A301-829A; RRID:AB_1264331
Rabbit polyclonal anti-TBK1	Bethyl	Cat#A300-093A; RRID:AB_2303002
Rabbit polyclonal anti-cleaved caspase 3	Cell Signaling	Cat#9661S; RRID:AB_2341188
Rabbit monoclonal anti-eIF2α (total)	Cell Signaling	Cat#D7D3; RRID:AB_10692650
Mouse monoclonal anti-puromycin (clone 12D10)	Merck Millipore	Cat#MABE343; RRID:AB_2566826
Rabbit polyclonal anti-FUS	Proteintech	Cat#11570-1-AP; RRID: AB_2247082
Rabbit polyclonal anti-Nup107	Proteintech	Cat#19217-1-AP; RRID:AB_10597702
Rabbit polyclonal anti-Nup98-Nup96	Proteintech	Cat#12329-1-AP; RRID:AB_10973678
Rabbit polyclonal anti-YBX1	Proteintech	Cat#20339-1-AP; RRID:AB_10665424
Rabbit polyclonal anti-ATXN2	Proteintech	Cat#21776-1-AP; RRID:AB_10858483
Rabbit polyclonal anti-KPNA2	Proteintech	Cat#10819-1-AP; RRID:AB_2265526
Rabbit polyclonal anti-TNPO1	Proteintech	Cat#20679-1-AP; RRID:AB_10694291
Mouse monoclonal anti-FUS	Santa Cruz	Cat#sc-47711; RRID:AB_2105208
Rabbit polyclonal anti-βIII-tubulin (Tuj)	Sigma-Aldrich	Cat#T2200; RRID:AB_262133
Mouse monoclonal anti-β-actin	Sigma-Aldrich	Cat#A5441; RRID:AB_476744
Bacterial and Virus Strains		
NEB 5-alpha Competent *E. coli*	New England Biolabs	Cat#C2987
Respiratory syncytial virus (RSV) wild-type strain A2	Openshaw Lab 18287232 (first used in this study)	N/A
Biological Samples		
Spinal cord tissue from ALS patients and healthy individuals (frozen)	Sheffield Brain Tissue Bank	Request No.15-011
Spinal cord tissue from ALS patients and healthy individuals (paraffin blocks)	MRC London Neurodegenerative Diseases Brain Bank	Request No.1470
Chemicals, Peptides, and Recombinant Proteins		
MG132 (proteasome inhibitor)	Calbiochem	Cat#474790
Pifithrin-μ (HSP70 inhibitor)	Enzo Life Sciences	Cat#BML-AP503
Polyinosinic–polycytidylic acid potassium salt, poly(I:C)	Sigma-Aldrich	Cat#P9582
Sodium arsenite	Sigma-Aldrich	Cat#35000
Dithiothreitol (DTT)	Sigma-Aldrich	Cat#43815
Interferon beta-1a	Sigma-Aldrich	Cat#IF014
Cycloheximide	Sigma-Aldrich	Cat#C7698
Puromycin dihydrochloride	Sigma-Aldrich	Cat#P8833
5,6-Dichlorobenzimidazole 1-β-D-ribofuranoside (DRB)	Sigma-Aldrich	Cat#D1916
Actinomycin D	Sigma-Aldrich	Cat#A1410
Critical Commercial Assays		
GenElute™ Total RNA Purification Kit	Sigma-Aldrich	Cat#RNB 100
RNase free DNase set	QIAGEN	Cat#79254
USB® Poly(A) Tail-Length Assay Kit	Thermo Scientific	Cat#764551KT

(*Continued on next page*)

Continued

REAGENT or RESOURCE	SOURCE	IDENTIFIER
FuGENE® HD transfection reagent	Promega	Cat#E2311
DreamTaq HS polymerase	Thermo Scientific	Cat#EP2702
SuperScript IV Reverse Transcriptase	Thermo Scientific	Cat# 18090010
Lipofectamine 2000 transfection reagent	Thermo Scientific	Cat#11668027
Experimental Models: Cell Lines		
Human: SH-SY5Y neuroblastoma cells (ATCC)	Sigma-Aldrich	Cat#94030304
Human: SH-SY5Y FUS line: ΔNLS1_ho (homozygous)	An et al., 2019	N/A
Human: SH-SY5Y FUS line: ΔNLS4_ho (homozygous)	An et al., 2019	N/A
Human: SH-SY5Y FUS line: ΔNLS7_ho (homozygous)	An et al., 2019	N/A
Human: SH-SY5Y FUS line: ΔNLS2_het (heterozygous, FUS granule positive)	An et al., 2019	N/A
Human: SH-SY5Y FUS line: ΔNLS8_het (heterozygous)	An et al., 2019	N/A
Human: SH-SY5Y FUS line: ΔNLS10_het (heterozygous)	An et al., 2019	N/A
Human: SH-SY5Y FUS line: ΔNLS11_het (heterozygous, FUS granule positive)	An et al., 2019	N/A
Human: SH-SY5Y FUS line: FUS knockout line	An et al., 2019	N/A
Human: primary fibroblasts (healthy control and bearing FUS P525L mutation)	Lo Bello et al., 2017	N/A
Human: Day 40 motor neurons derived from hES H9 cells	Shelkovnikova et al., 2017	N/A
Oligonucleotides		
Primers for qRT-PCR, see Table S1	An et al., 2019 and this paper	N/A
Primer: Poly(A) tail assay for FUS, P1: 5′-GTCCAGCCCA TGTGAGACTT-3′	This paper	N/A
Primer: Poly(A) tail assay for FUS, P2: 5′-AACCTCCAGC ATAAAAGGGCT-3′	This paper	N/A
siRNA, targeting human STAT1: MISSION® esiRNA	Sigma-Aldrich	Cat#EHU010121
siRNA, scrambled: AllStars Negative Control siRNA	QIAGEN	Cat#SI03650318
Recombinant DNA		
Plasmid: FUS R522G GFP	Shelkovnikova et al., 2014a	N/A
Software and Algorithms		
CellF software	Olympus	N/A
Zen software	Zeiss	https://www.zeiss.com/microscopy/int/products/microscope-software/zen.html
ImageJ	Schneider et al., 2012	https://imagej.nih.gov/ij/
Adobe Photoshop CS3	Adobe Inc.	https://www.adobe.com/uk/products/photoshop.html
GraphPad Prism 6	GraphPad Software, Inc.	https://www.graphpad.com/scientific-software/prism/
Leica Application Suite AF software	Leica Microsystems	https://www.leica-microsystems.com/products/microscope-software/p/leica-application-suite/
Other		
BX61 microscope equipped with F-View II camera	Olympus	https://www.olympus-ims.com/en/microscope/bx61-2/
LSM880 microscope	Zeiss	https://www.zeiss.com/microscopy/int/products/confocal-microscopes.html
EVOS XL Core system	Thermo Scientific	Cat#AMEX1100
Leica TCS SP2 MP confocal microscope	Leica Microsystems	https://www.leica-microsystems.com/products/confocal-microscopes/p/leica-tcs-sp2/
StepOnePlus™ Real-Time PCR System	Thermo Scientific	Cat#4376600

LEAD CONTACT AND MATERIALS AVAILABILITY

Further information and requests for resources and reagents should be directed to and will be fulfilled by the Lead Contact, Tatyana Shelkovnikova (shelkovnikovat@cardiff.ac.uk). All reagents generated in this study are available from the Lead Contact and may require a completed Materials Transfer Agreement.

EXPERIMENTAL MODEL AND SUBJECT DETAILS

Cell lines

Parental SH-SY5Y human neuroblastoma cell line (originating from a 4-year old female) was obtained from Sigma (94030304). Generation and characterization of FUSΔNLS SH-SY5Y cell lines has been described in earlier (An et al., 2019). Human fibroblasts acquisition was approved by the University of Palermo Review Board (prot.07/2017). Characterization of fibroblasts from a female patient with FUS P525L mutation was described earlier (Chiò et al., 2009; Lo Bello et al., 2017). SH-SY5Y cells and human fibroblasts were grown in high-glucose 1:1 mixture of DMEM/F12 supplemented with 10% fetal bovine serum (FBS), 1% penicillin-streptomycin and 2 mM L-glutamine (all Invitrogen). Human motor neuron differentiation from ES cells (H9 line, female) is described in a previous study (Shelkovnikova et al., 2017).

Virus strain

RSV wild-type strain A2 (18287232) was used at the multiplicity of infection (MOI) of 10 for infecting neuroblastoma cells and human fibroblasts. Inoculation was performed in normal culturing medium.

Human post-mortem tissue

Human samples from clinically and histopathologically characterized ALS cases and healthy individuals were obtained from the Sheffield Brain Tissue Bank and MRC London Neurodegenerative Diseases Brain Bank. Consent was obtained from all subjects for autopsy, histopathological assessment and research were performed in accordance with local and national Ethics Committee approved donation. Four ALS-FUS patients were included in the study: male (R521C mutation); female (R521C mutation); male (R524T mutation); and male (G507N mutation). Immunohistochemistry analysis was performed as described earlier (Shelkovnikova et al., 2014b).

METHOD DETAILS

Cell transfection and treatments

Transfections were performed with 200 ng plasmid DNA, 250 ng poly(I:C) (Sigma) or siRNA in 24-well plates using Lipofectamine 2000 (Invitrogen). hES cell derived motor neurons were transfected with 1 μg poly(I:C) using FuGENE® HD reagent (Promega). Final concentrations of compounds were as follows: 0.5 mM sodium arsenite (Sigma), 1 mM DTT (Sigma), 50 μM MG132 (Calbiochem), 10 μg/ml puromycin (Sigma), 5 μM pifithrin-μ (Enzo Life Sciences), 10 μg/ml cycloheximide (Sigma), 5 μg/ml actinomycin D (Sigma) and 25 μg/ml DRB (Sigma). Cells were treated with $1x10^4$ IU Interferon beta-1a (Sigma) for the indicated periods of time.

Immunocytochemistry, light and confocal microscopy

Cells grown on coverslips were fixed in 4% paraformaldehyde and permeabilised with methanol. Primary antibodies diluted in blocking buffer (5% goat serum in 0.1% Tween-20/PBS) were added on cells for 1 h at RT or overnight at 4°C. Secondary antibodies in 0.1% Tween-20/PBS (Alexa488- or Alexa546-conjugated, Invitrogen) were applied for 1 h at RT. DAPI was used to visualize nuclei. To detect $poly(A)^+$ mRNA, cells were incubated at 37°C with 1 μM Cy5-labeled oligo(dT)30 probe (Sigma) diluted in hybridization buffer (2x SSC, 25% formamide) overnight, followed by anti-G3BP1 immunostaining. Fluorescent images were obtained using BX61 microscope equipped with F-View II camera and CellF software (all Olympus). Confocal fluorescent images were taken using LSM880 microscope with ZEN software (Zeiss). Plot Profile and 3D Surface Plot functions of ImageJ were used to create profile/surface plots of protein colocalization. Bright-field images of human fibroblasts were taken using EVOS XL Core system. Quantification of stress granules and cleaved caspase 3 positive cells was performed using 'Analyze particles' function of ImageJ software. Images were assembled using Photoshop CS3. For live imaging of FUS aggregate assembly, SH-SY5Y cells were cultured on glass-bottomed culture dishes (Mattek) and transfected with a plasmid for the expression of GFP-tagged FUS R522G. After 24 h, cells were transfected with poly(I:C) and maintained in HEPES-buffered medium during imaging. Imaging was carried out using a Leica TCS SP2 MP confocal microscope (Fluotar L 63 × 1.4 oil objective) equipped with an on-scope incubator with temperature control (Leica Microsystems). A sequence of images taken with 7 min intervals was subsequently converted into a movie using Leica Application Suite AF software.

RNA extraction, qRT-PCR and poly(A)-tail (PAT) length assay

Total RNA was extracted from cells using GenElute Total RNA Purification Kit (Sigma) and possible DNA contamination was eliminated with RNase free DNase kit (QIAGEN). Total RNA (500 ng) was used for first-strand cDNA synthesis with Superscript IV

(Invitrogen). qPCR was performed on StepOne Plus RT-PCR System using DreamTaq HS polymerase (Thermo Scientific), and gene expression was normalized to that of GAPDH. Poly(A)-tail length assay was carried out using USB® Poly(A) Tail-Length Assay Kit (Thermo Scientific) as instructed. PCR products were resolved on 3% agarose gel and lane intensity was plotted in arbitrary units using Plot Profile function of ImageJ.

Western blotting

Cell were lysed on plates in 2xLaemmli buffer and the lysates were boiled at 100°C for 5 min. Proteins were separated on 10% SDS-PAGE and transferred to PVDF membranes followed by incubation in 4% skimmed milk for 1 h and in primary antibodies (1:1,000) at 4°C overnight. HRP-conjugated secondary antibodies (1:3,000, Amersham) were applied at RT for 1.5 h. ECL solution (Advansta) was used for chemiluminescent detection. Western blots were re-probed for β-actin as a control for equal loading. Protein labeling with puromycin was performed as described earlier (Shelkovnikova et al., 2017).

QUANTIFICATION AND STATISTICAL ANALYSIS

Statistical analysis was carried out using GraphPad Prism 6 software. Mean values of biological replicates were compared using appropriate tests (stated in the figure legends). Significance levels are indicated with asterisks (*$p < 0.05$, **$p < 0.01$, ***$p < 0.001$, ****$p < 0.0001$). N indicates the number of biological replicates. Error bars represent standard error of the mean (SEM).

DATA AND CODE AVAILABILITY

This study did not generate any unique datasets or codes.

Cell Reports, Volume *29*

Supplemental Information

Antiviral Immune Response as a Trigger of FUS Proteinopathy in Amyotrophic Lateral Sclerosis

Tatyana A. Shelkovnikova, Haiyan An, Lucy Skelt, John S. Tregoning, Ian R. Humphreys, and Vladimir L. Buchman

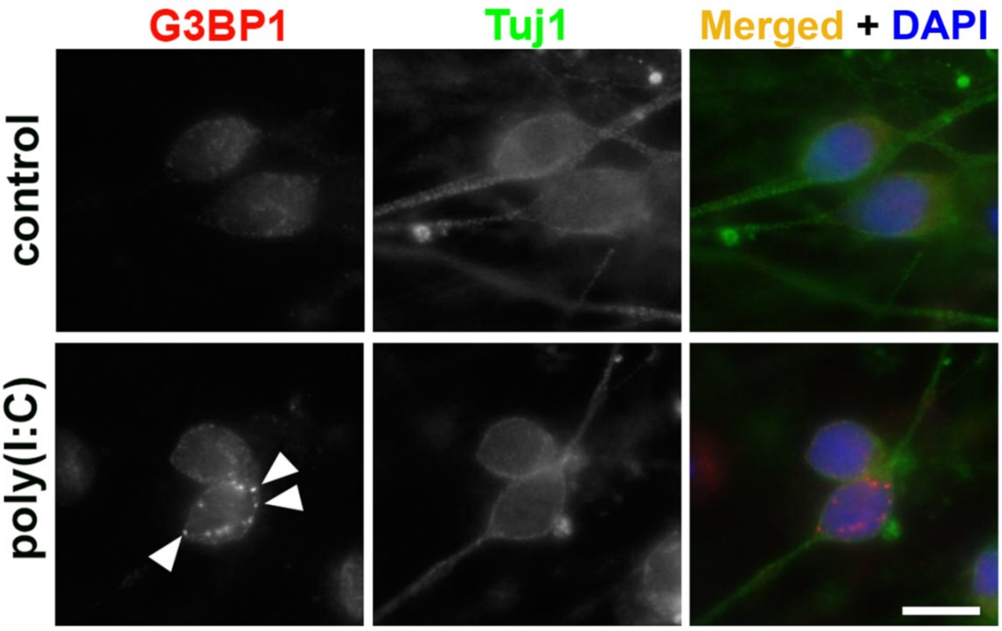

Figure S1. Poly(I:C) transfection is capable of inducing SGs in human embryonic stem (ES) cell derived motor neurons, Related to Figure 1.

DIV40 neurons were analysed 8 h post-transfection with poly(I:C). Arrowheads point to G3BP1-positive SGs. Scale bar, 10 µm.

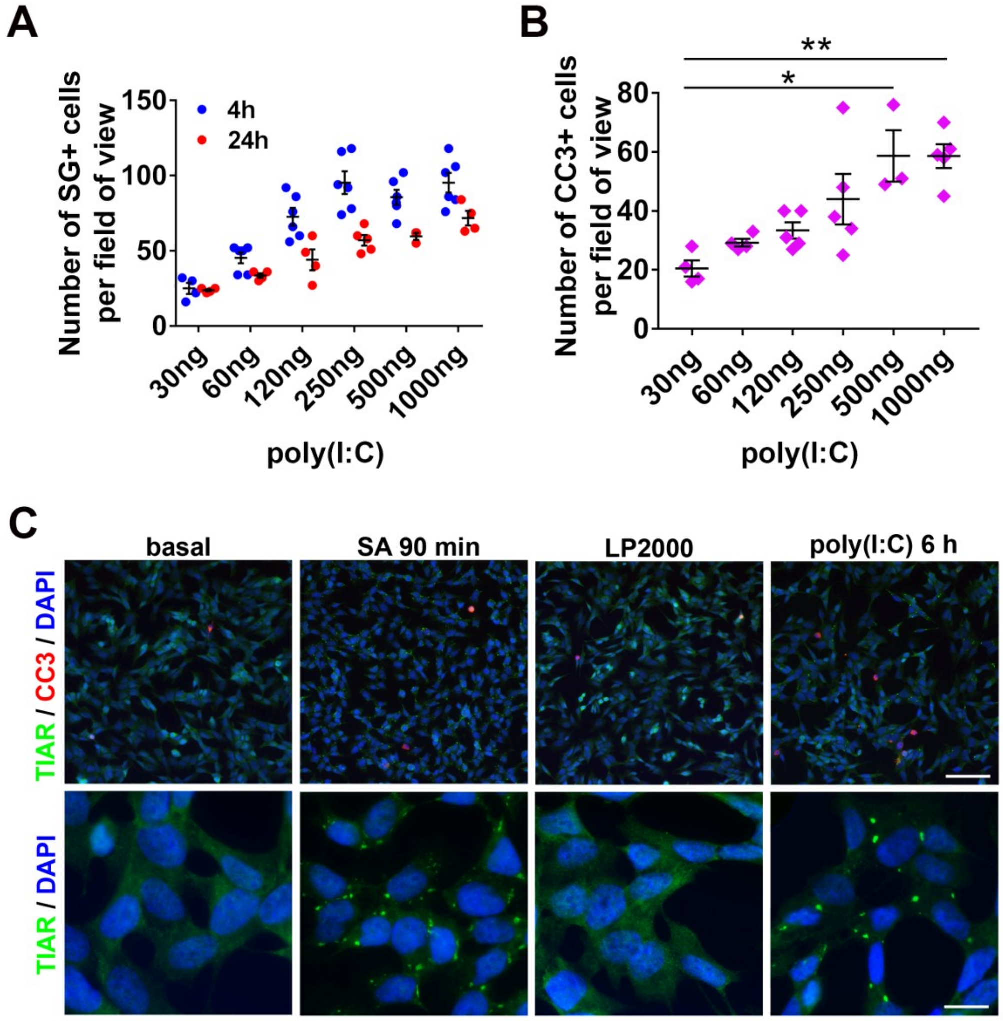

Figure S2. Concentration-dependent ability of poly(I:C) to induce SGs and toxicity in SH-SY5Y cells, Related to Figure 1.

(A) Concentration-dependent induction of SGs by poly(I:C) in SH-SY5Y cells. Cells were transfected with the corresponding amount of poly(I:C) (per well, in 24-well plates) and analysed 4 and 24 h post-transfection using anti-TIAR staining (x40 magnification). Data are represented as mean±SEM.

(B) Concentration-dependent toxicity of poly(I:C) in SH-SY5Y cells. Cells were transfected with the corresponding amount of poly(I:C) (per well, in 24-well plates) and analysed 24 h post-transfection using anti-cleaved caspase 3 (CC3) staining (x20 magnification). Note that 250 ng was selected for all subsequent experiments due to the optimal balance between SG induction and cell survival. Data are represented as mean±SEM. $^*p<0.05$, $^{**}p<0.01$ (Mann-Whitney *U* test).

(C) Poly(I:C) stimulation for 6 h and SA treatment for 1.5 h do not affect cell morphology and do not induce significant toxicity. SH-SY5Y cells were treated with SA or transfected with poly(I:C) and analysed after 1.5 h or 6 h, respectively, using a combination of anti-TIAR and cleaved caspase 3 (CC3) staining. Lipofectamine2000 (LP2000)-treated cells were included as a control for poly(I:C) transfection. Scale bars, general plane – 100 μm, close up – 10 μm.

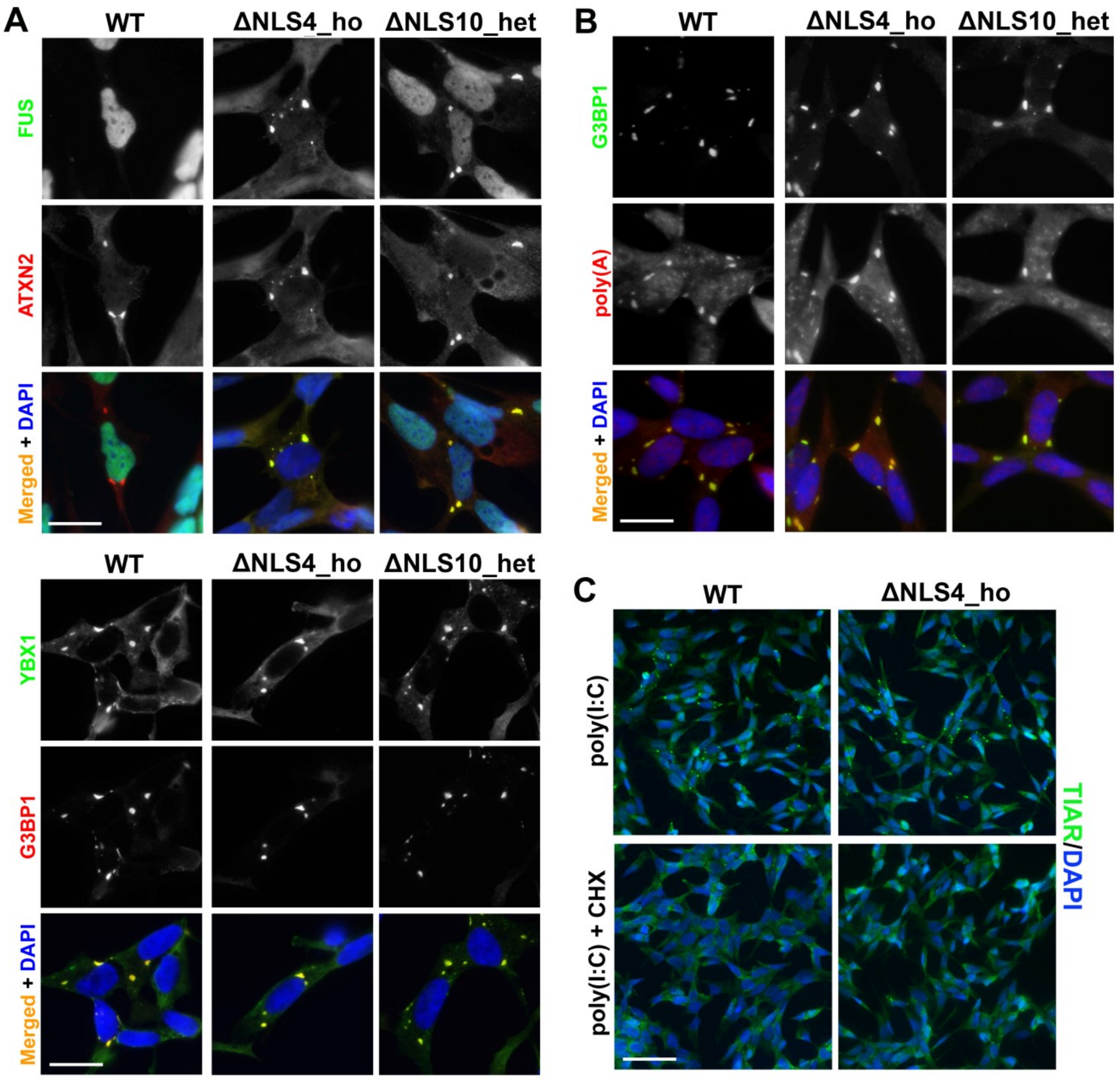

Figure S3. Poly(I:C)-induced SGs, including those in FUSΔNLS cells, contain SG markers ATXN2 and YBX1 (A) as well as polyadenylated RNA (B), and are sensitive to cycloheximide treatment (C), Related to Figure 2.

Cell lines were transfected with poly(I:C) and analysed 4 h post-transfection. In C, cycloheximide (CHX) was added 1 h prior poly(I:C) transfection. Representative images are shown. Scale bar, A, B – 10 μm, C – 50 μm.

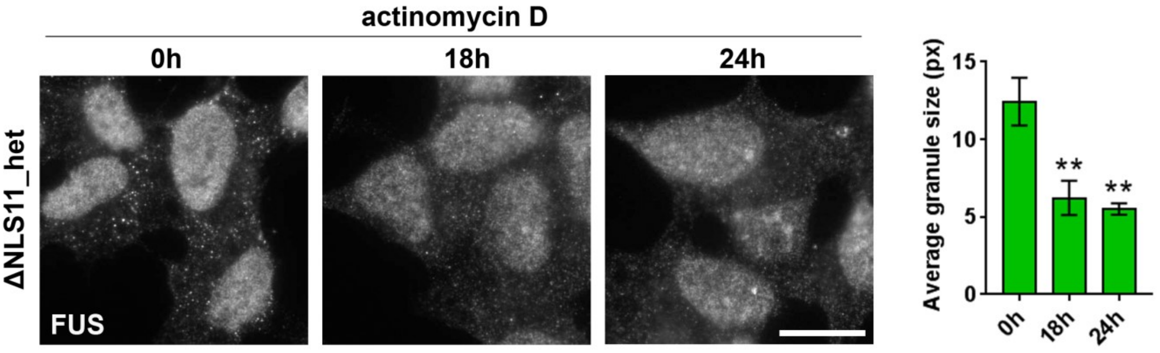

Figure S4. Spontaneous FUS granules formed by endogenous mutant FUS protein (endoFGs) are sensitive to transcription inhibition, Related to Figure 3.

ΔNLS11_het line which possesses endoFGs was treated with actinomycin D for 18 h and 24 h, and the size of FGs was measured using Image J (px – pixels). Data are represented as mean±SEM. **p<0.01 (ANOVA with Dunn's test). Scale bar, 10 μm.

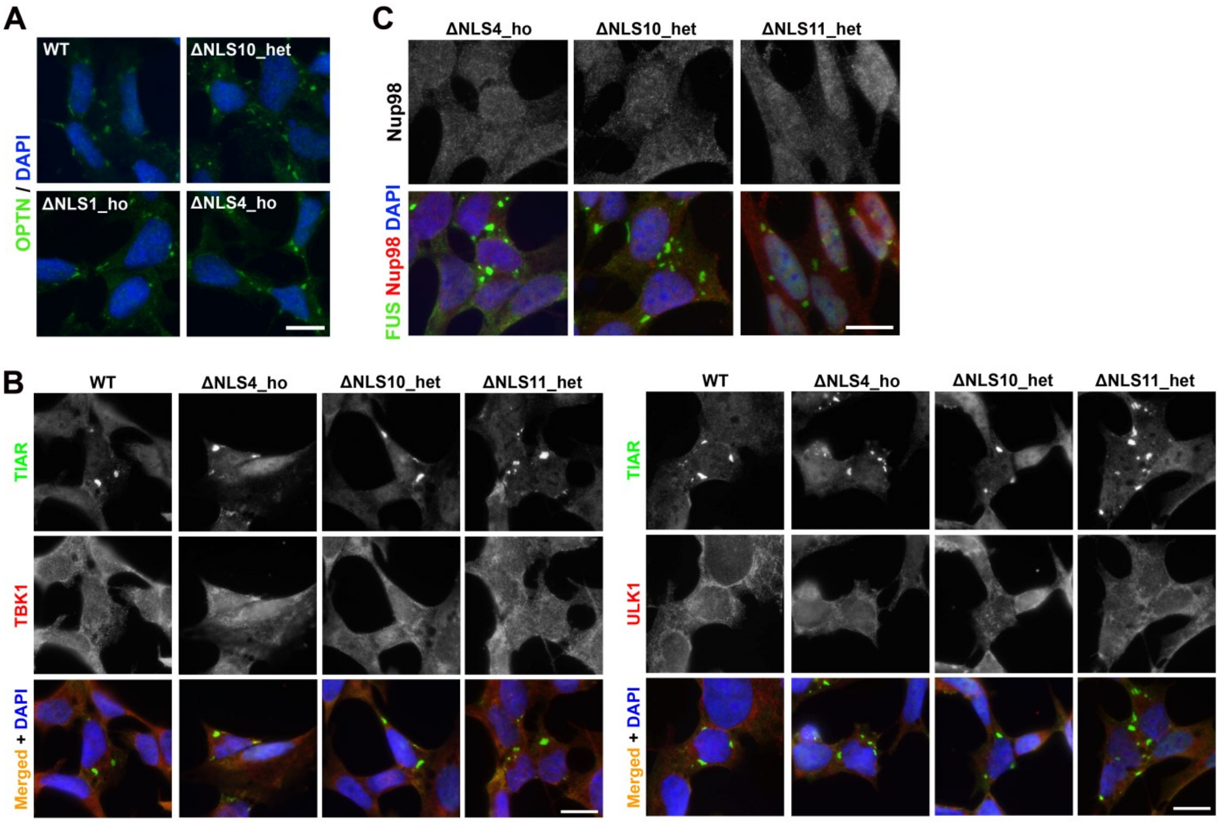

Figure S5. Analysis of sequestration of autophagy-related proteins and NPC factors into mutant FUS assemblies, Related to Figure 4.

(A) Optineurin is recruited into SA-induced SGs in WT and FUSΔNLS cells. Cells were treated with SA for 1 h.

(B) Optineurin interactor TBK1 and the primary component of the autophagy initiation complex ULK1 are not detectable in mutant FUS-containing cytoplasmic assemblies in poly(I:C)-stimulated FUSΔNLS cells.

(C) Nucleocytoplasmic transport factor Nup98 is not detected in mutant FUS-containing cytoplasmic assemblies in poly(I:C)-stimulated FUSΔNLS cells.

In B and C, cells were analysed 6 h post-transfection; note that ΔNLS11_het cell line contains endoFGs and therefore forms FAs not SGs.

Scale bars, 10 μm.

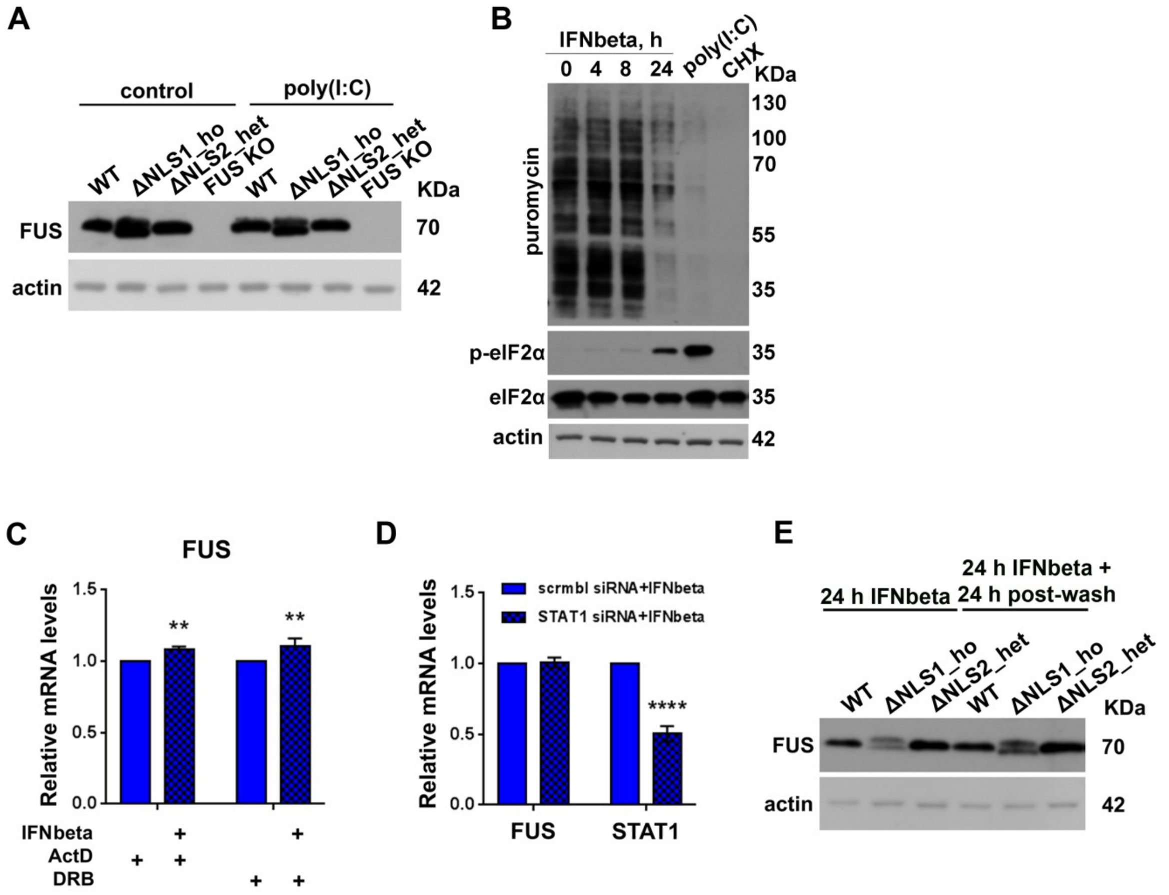

Figure S6. IFN-beta causes accumulation of FUS protein, Related to Figure 6.

(A) Poly(I:C) transfection does not result in increased FUS protein level in WT or FUSΔNLS cells. Cells were analysed at 24 h post-transfection.

(B) Puromycin labelling of proteins reveals significant impairment of protein translation due to eIF2α phosphorylation in poly(I:C)-transfected cells but not in IFN-beta treated cells. Cells were treated with IFN-beta and collected at the indicated time-points, or collected 8 h after poly(I:C) transfection. Cells treated with a protein synthesis inhibitor cycloheximide (CHX) were included as a negative control.

(C) Transcription inhibition does not prevent IFN-beta induced FUS mRNA accumulation. Cells were pre-treated with transcription inhibitors actinomycin D or DRB for 1 h. FUS mRNA level was measured 4 h into IFN-beta treatment by qRT-PCR. Data are represented as mean±SEM. N=4, **$p<0.01$ (Mann-Whitney *U* test).

(D) FUS mRNA upregulation in IFN-beta treated cells is independent of STAT1. Cells were transfected with scrambled siRNA or STAT1 siRNA; 48 h post-transfection, cells were subjected to IFN-beta for 4 h, and FUS mRNA level was measured by qRT-PCR. Data are represented as mean±SEM. N=4, ****$p<0.0001$ (Mann-Whitney *U* test).

(E) FUS protein continues to accumulate after IFN-beta wash-off both in WT and FUSΔNLS cells. Cells were treated with IFN-beta for 24 h, washed and analysed after another 24 h.

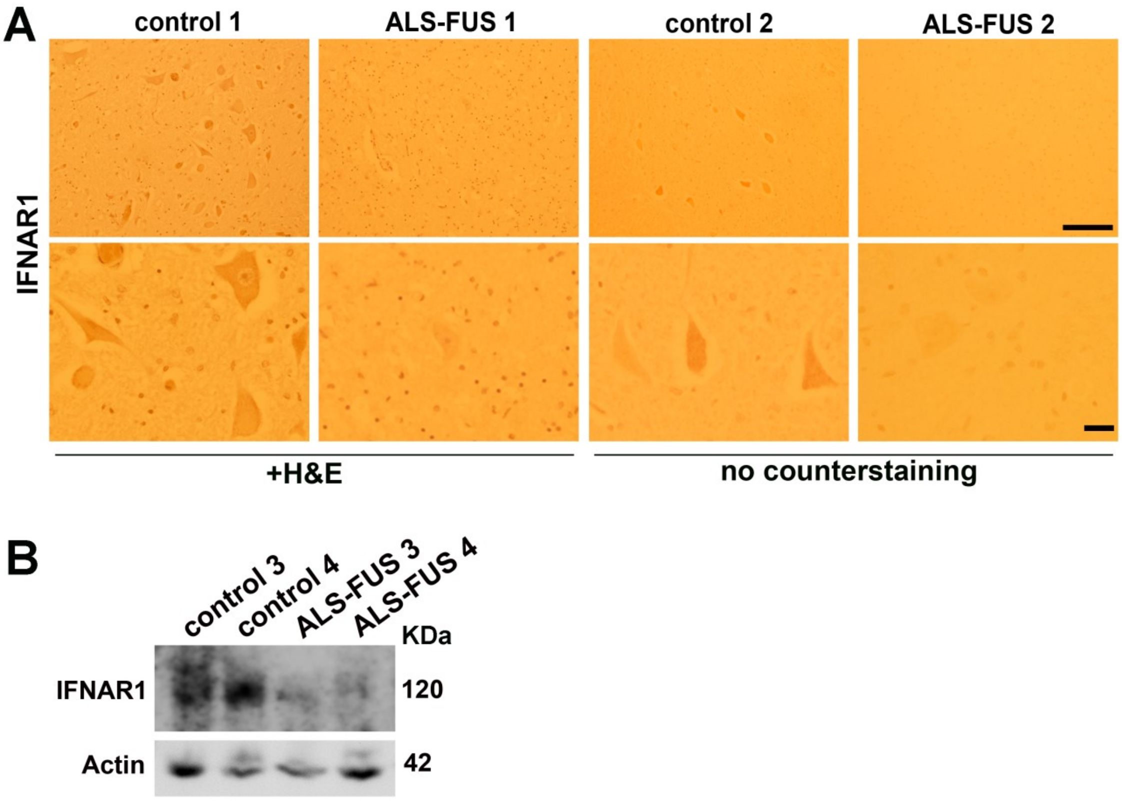

Figure S7. IFNAR1, one of the two IFN receptor (IFNAR) subunits, is highly expressed in spinal motor neurons and is downregulated in ALS-FUS, Related to Figure 6.

(A) IFNAR1 immunohistochemistry in the spinal cord sections for two control cases and two ALS-FUS cases. Note that control 1 and ALS-FUS 1 were counter-stained with H&E. Scale bars, 50 μm and 10 μm in upper and lower panels, respectively.

(B) Western blot analysis of IFNAR1 levels in the spinal cord of two control and two ALS-FUS patients.

Table S1. Primers for qRT-PCR used in the study, Related to STAR Methods.

Target and primer sequence	Source	ID
GAPDH, forward primer: 5'-TCGCCAGCCGAGCCA-3'	An et al., 2019	N/A
GAPDH, reverse primer: 5'-GAGTTAAAAGCAGCCCTGGTG -3'	An et al., 2019	N/A
FUS, forward primer: 5'-GGAACTCAGTCAACTCCCCA-3'	An et al., 2019	N/A
FUS, reverse primer: 5'-TACCGTAACTTCCCGAGGTG-3'	An et al., 2019	N/A
FUS ex7-, forward primer: 5'-CAGAGGTGGCATGGGGC-3'	This paper	N/A
FUS ex7-, reverse primer: 5'-TGTAACATTCTCACCCAGGC-3	This paper	N/A
FUS pre-mRNA, forward primer1: 5'-GAACCACCTCCAGAAAGGGG-3'	This paper	N/A
FUS pre-mRNA, reverse primer1: 5'-TGGGGCAAACCCATTTGGTA-3'	This paper	N/A
FUS pre-mRNA, forward primer2: 5'- GAAGCCGCGGAGAAGAGTAA-3'	This paper	N/A
FUS pre-mRNA, reverse primer2: 5'- AAGAAAAGACTTCCCGCCCC-3'	This paper	N/A
STAT1, forward primer: 5'-CTGTGCGTAGCTGCTCCTTT-3'	This paper	N/A
STAT1, reverse primer: 5'-GGTGAACCTGCTCCAGGAAT-3'	This paper	N/A
IFN-beta, forward primer: 5'-ACGCCGCATTGACCATCTAT-3'	This paper	N/A
IFN-beta, reverse primer: 5'-AGCCAGGAGGTTCTCAACAA-3'	This paper	N/A
IFIT3, forward primer: 5'-AGAGACACAGAGGGCAGTCA-3'	This paper	N/A
IFIT3, reverse primer: 5'-AAGTTCCAGGTGAAATGGCA-3'	This paper	N/A
CXCL10, forward primer: 5'-AAGTTCCAGGTGAAATGGCA-3'	This paper	N/A
CXCL10, reverse primer: 5'-ATGCTGATGCAGGTACAGCG-3'	This paper	N/A
CHOP, forward primer: 5'-TTAAAGATGAGCGGGTGGC-3'	Shelkovnikova et al., 2017	N/A
CHOP, reverse primer: 5'-GCTTTCAGGTGTGGTGATGTA-3'	Shelkovnikova et al., 2017	N/A
